# Community Culinary Workshops as a Nutrition Curriculum in a Preventive Medicine Residency Program

**DOI:** 10.15766/mep_2374-8265.10859

**Published:** 2019-12-13

**Authors:** Ryan D. Lang, Mary Carol Jennings, Clarence Lam, Hsin-Chieh Yeh, Colin Zhu, Tina Kumra

**Affiliations:** 1 Instructor, Department of Health Policy and Management, Johns Hopkins University School of Public Health; 2 Assistant Scientist, Department of Health Policy and Management, Johns Hopkins University School of Public Health; 3 Associate Professor, Department of Medicine, Johns Hopkins University School of Medicine; 4 Physician Chef-Consultant, Independent Practice; 5 Assistant Professor, Department of Pediatrics, Johns Hopkins University School of Medicine

**Keywords:** Culinary Medicine, Nutrition, Community-Based Participation, Community-Based Medicine, Preventive Medicine

## Abstract

**Introduction:**

Obesity and diabetes are common diagnoses in the primary care population, especially in urban settings. Physicians providing preventive culinary and nutrition education to patients may be able to uniquely address these medical issues; however, culinary and nutrition education among medical residency programs is insufficient.

**Methods:**

We describe a pilot of a novel interactive approach to culinary and nutrition education focused on preventive medicine residents who were trained to provide culinary and nutrition skills to community members in three separate workshops. We developed and implemented a series of three culinary education workshops with 11, eight, and nine preventive medicine residents in each respective workshop. A total of 16 residents were invited to participate. A physician-chef facilitated each workshop with the residents within a community church kitchen and meeting area. We evaluated self-reported data on confidence level with culinary education and resident attitudes toward effects of culinary education on patient behaviors, as well as frequency of home-cooked meals and personal cooking competency, as indicators of resident proficiency.

**Results:**

A significant increase was noted in self-reported cooking competency after culinary workshops when evaluating change from the first workshop to the final workshop ( *p* = .038). Increases in home-cooking frequency and belief that lifestyle medicine impacts patient behavior were also observed but did not achieve statistical significance.

**Discussion:**

Culinary workshops are a useful tool to enhance nutrition education in a residency curriculum and may be an effective way to improve resident perceptions regarding the impact of nutrition education in the community.

## Educational Objectives

By the end of this activity, learners will be able to:

1. Increase their own culinary skills and home-cooking behaviors.
2. Discuss practical culinary skills and nutritional counseling relevant to patients in an underserved setting.
3. Demonstrate culinary skills and nutritional counseling in a community kitchen.

## Introduction

Diet and nutrition have a major impact on health and the development of chronic diseases.^1^ Poor diet accounted for 529,299 deaths in 2016 and contributed to 14% of total disability-adjusted life years in 2010.^2^ Nutrition education is limited in medical curricula, and nearly 80% of medical school faculty in one study reported that students need additional nutrition instruction.^3^ The same study showed that just over a quarter of US medical schools offer the minimum number of 25 hours in nutrition education recommended by the National Academy of Sciences.^3,4^ Opportunities for community engagement among medical trainees outside of the health care system have traditionally been limited; however, utilizing service learning in the context of nutrition education may provide valuable educational benefits to medical trainees while simultaneously improving the health of community members.^5^

Culinary and nutrition education for health care providers through training conferences and presentations may be associated with greater cooking frequency among participants and may lead to more effective nutritional counseling for obese and overweight patients.^6^ Some preventive medicine residency programs have begun curricular implementation of lifestyle medicine core competencies—some of which include holistic and alternative approaches to patient wellness.^7^ A primary care curriculum for physician nutrition education that includes offering conference meals illustrating nutrition lecture topics, as well as brief taste tests not in conjunction with lectures, has been described previously,^8^ and the number of culinary education programs for physicians, as evidenced by a recent report that lists physician and medical trainee culinary education programs, is growing.^9^ There are no publications, however, that describe the process of integrating culinary education training using community workshops into the preventive medicine curriculum.^6,10^

Our preventive medicine residency program and patient-centered medical home (PCMH) practitioners within our health system developed a novel resident-run preventive medicine consult service serving the Remington neighborhood of Baltimore, Maryland. These healthy lifestyle clinical services and patient counseling services were identified by administrative review as being popular among patients. Yet, as seen in related reviews of primary care residency training programs, baseline review of our residency training curriculum showed that residents reported relatively low confidence in providing nutrition-based care, counseling, and behavior modification.^11^ The structure and format of primary care clinics typically do not allow sufficient opportunities for residents to develop these skills. Based on Kolb's theoretical framework on experiential learning, there are four stages of learning: concrete learning, reflective observation, abstract conceptualization, and active experimentation.^12^ We developed a nutrition and culinary curriculum that allowed for a focus on the first two stages of this learning cycle in a neighborhood teaching kitchen through interactive demonstrations led by a physician-chef.

Clinicians who practice healthy behaviors, such as exercising or consistently wearing a seat belt, are more likely to counsel patients on these preventive behaviors.^13^ This curriculum aimed to provide community-based training for preventive medicine residents to increase their own cooking confidence and behaviors and secondarily to increase their confidence in delivering nutrition counseling to the community.^10^ Providing these skills in a workshop format allowed for active cooking, group discussion, and eating cooked meals together after workshops, which have been shown in previous studies to increase fruit and vegetable consumption among participants.^13^ In the long term, an increase in self-reported confidence may enable residents to better counsel and transfer knowledge and skills for healthy food selection and preparation to patients and community members.^6,13^ By holding the workshops in a community kitchen setting, we created a more realistic venue for the resident trainees that would help them understand the resources, food, and culture of their patients.

Recent review of nutrition education literature has focused on a diverse array of interventions to incorporate nutritional education in an already crowded medical curriculum.^14^ These interventions were mainly targeted toward medical students, and none focused on residents. In addition, the majority of these interventions were classroom based, and only a few had a community component.^14^ Our work adds to the existing education literature by utilizing an interactive approach to nutritional counseling and culinary skills for resident physicians and by using a curriculum created in conjunction with the patient community being served. This pilot can inform residency faculty who wish to create curricula that enhance nutrition education for residents and encourage greater interaction with community members, specifically in high-need areas.

## Methods

We developed a series of culinary medicine workshops for a 2-year cohort of general preventive medicine residents. Instructional methods included PowerPoint presentations and interactive demonstrations involving residents in the kitchen setting. Residents attended three workshops each for 4-hour periods in the community kitchen and meeting space of a neighborhood church in the Remington neighborhood of Baltimore from July 2016 to April 2017. There was no prerequisite knowledge needed by residents prior to engaging in culinary medicine workshops; however, residents were asked to bring their own chef knives to each workshop. The residency program obtained funding to provide recipe ingredients and utilized the existing resources of the community kitchen, making adjustments as needed to the recipes according to the availability of affordable ingredients and appliances.

The workshop kitchen was industrial sized, with three oven ranges and a large island, and a large meeting room adjacent to the kitchen was used for didactic sessions, social gathering and eating, and workshop debriefings. We selected the church because of its proximity to the aforementioned PCMH with the health system. Some community and church members used this clinic as their primary care clinic.

We cocreated the learning objectives of the workshop curriculum through a participatory process including planning discussions with community members from the neighborhood surrounding the church, as well as a literature review. We then consulted with the physician-chef facilitator to develop a final workshop curriculum for the residents focused on basic skills that could easily be learned and used by residents to subsequently train and counsel community members. The learning objectives of the first workshop were to discuss the importance of cooking for health, provide a kitchen orientation, demonstrate basic knife skills, and list safe and unsafe behaviors in the kitchen. The learning objectives of the second workshop were to list 30 basic ingredients of a well-stocked kitchen, name the general price range of healthy pantry staples, and discuss the use of appliances. The learning objectives of the third workshop were to demonstrate proper skills and knowledge to read recipes and labels on food, describe the basic elements of a well-balanced plate, and discuss strategies that could be used to purchase healthy foods in a price-sensitive manner.

The physician-chef who led the workshops was a primary care physician, board certified in family medicine and trained as a professional chef and health educator. Although the physician-chef was a visiting faculty member, all other faculty involved with workshop development were core faculty members within our preventive medicine residency program. Two facilitators were used in each workshop in addition to the physician-chef to help set up workstations and answer participant questions. One was a program administrator for the program with no culinary training, and the other was a preventive medicine doctor with no formal culinary training. Both facilitators were faculty members with our residency program. We also enlisted support from select church members, including the church pastor, to participate in resident workshops and provide informal feedback. The ideal number of participants for each workshop was 12, and kitchen workstations were set up to allow for teams of three to work together.

We started each workshop with introductions that allowed participants to identify food preferences and allergies, as well as share their level of experience in the kitchen (Appendix A provides a full schedule and facilitator guide). The physician-chef then led didactics that helped cover the learning objectives of the workshop using PowerPoint slides (please see Appendices B–D). Next, the participants were oriented to the kitchen and assigned to workstations in groups of three to four. Customizable plant-based recipes low in saturated fat and sodium were used to complement the curriculum during each of the three workshops, and the workshop facilitator coached residents through preparation and teaching techniques (please see Appendices E–G). Meat and fish were excluded from the baseline recipes to offer exposure to plant-based meals, and a concerted effort was made to choose recipes that were low in saturated fat content. While eating, the facilitators helped lead a reflection session with core questions included in Appendix A, including perceived challenges in making the recipe and receptiveness of community members to these recipes. Finally, the participants assisted with cleaning up and asked any final questions.

At the end of each workshop, participating residents were invited to complete a voluntary 19-item questionnaire including two demographic questions and 17 additional questions (Appendix H). Residents reported their self-perceived cooking expertise, how often they cooked meals at home, and their beliefs regarding the impact of culinary medicine education on patient health outcomes using a 5-point Likert scale. The primary outcomes of the program evaluation were (1) frequency of resident home-cooked meals and (2) resident self-assessment of cooking competency. The secondary outcomes of the program evaluation were (1) self-reported level of confidence in providing nutrition education and counseling and (2) resident attitude toward nutrition counseling. This questionnaire was not formally validated for content or construct validity.

Residents were given credit toward completion of residency program–required clinical education sessions for participation in culinary education workshops. Residents who were unable to attend scheduled workshops were allowed to participate in weekly community cooking classes to obtain credit, although they were not eligible to complete related postworkshop questionnaires and were not included in the study.

A message indicating the purpose of the culinary education workshops was provided with each questionnaire. The Johns Hopkins University Institutional Review Board provided a waiver for this study (ID No. IRB00107676) on August 1, 2016. Residents provided informed consent before participating in the study. Residents completed questionnaires using pen and paper and were asked not to provide self-identifiable information to protect anonymity.

We calculated the median (range) and proportions for each questionnaire item. We compared differences of responses between the initial workshop and the final workshop using Fisher's exact test. Because we did not collect personal identifiers, pre- and postworkshop data were not linked, and we did not perform paired tests. All analyses were performed using Stata Version 13 (StataCorp, College Station, Texas).

## Results

Demographic data of all residents in the program are noted in Table 1. Sixteen individual residents enrolled in the program during the 2016–2017 academic year, and all were invited to participate. Eleven residents (69% of the total residents in the program) attended the initial workshop, eight residents (50% of the total residents) attended the second workshop, and nine residents (56% of the total residents) attended the third and final workshop. Nine residents (56% of the total residents) attended two workshops, and five residents (31% of the total residents) attended all three workshops.

**Table 1.** Preventive Medicine Resident Demographic Data

Demographic	Residents (*N* = 16)
Age: median (range)	32.5 (20)
Female gender: *n* (%)	4 (25%)
Underrepresented in medicine: *n* (%)	5 (31%)
Another residency: *n* (%)	8 (50%)
PGY 2: *n* (%)	9 (56%)
PGY 3: *n* (%)	7 (44%)

The median age of residents was 32.5 years (29–49 years). Of the residents, 25.0% were female, 50.0% had completed another clinical residency prior to preventive medicine residency training, and 31.3% were from groups underrepresented in medicine (see Table 1). Residents who completed any of the three workshops were included in the analysis.

Among the residents who completed the workshops and questionnaires (11 residents in the initial workshop and nine residents in the final workshop), we observed an increase in residents self-reporting as “competent” cooks, with 50% of residents in the initial workshop providing this self-designation compared to 89% of residents in the final workshop, which is a statistically significant increase when using Fisher's exact test ( *p* = .038; Table 2). We observed small, nonsignificant increases in the proportion of residents who believed that nutritional counseling would translate to patients making moderate changes in eating behavior and in the proportion of residents who believed that integrative and lifestyle medicine among providers affects care from the initial workshop to the final workshop (see Table 2). There was no difference, however, in residents’ self-assessed comfort with providing nutritional or cooking counseling to patients.

**Table 2.** Summary of Self-Reported Skill Assessment Among Residents Completing Initial and Final Workshops

	Initial Workshop		Final Workshop		
	(*n* = 11)		(*n* = 9)		
Questionnaire Item	No.	%	No.	%	*p*
Primary outcomes					
“Intermediate” or “Expert” personal cooking confidence	6	50	6	67	1.000
“I myself am a competent cook”^a^	6	50	9	100	.038
“How many times per week do you eat home-cooked meals?” (Only five or more meals)	7	64	6	67	.920
“How many times per week do you cook at home on average?” (Only five or more meals)	4	33	4	44	1.000
“How many fruits per day do you eat on average?” (Only five or more fruits)	1	9	0	0	1.000
“How many vegetables per day do you eat on average?” (Only five or more vegetables)	2	18	1	11	.979
Secondary outcomes					
“I feel comfortable counseling patients about cooking, eating, and nutrition.”	8	73	7	78	1.000
“Most patients will try to change their lifestyle if I advise them to do so.”	5	45	4	44	1.000
“Physicians can have an effect on a patient's dietary behavior if they take the time to discuss the problem.”	7	58	7	78	.622
“Nonphysician clinic staff can have an effect on a patient's dietary behavior if they take time to discuss the problem.”	0	0	0	0	1.000
“For most patients, health education does little to promote adherence to a healthy lifestyle.”	0	0	1	11	.185
“My patient-education efforts will be effective in increasing patients’ compliance with nutritional recommendations.”	4	33	3	33	.657
“After receiving nutrition counseling, patients with poor eating habits will make moderate changes in their eating behavior.”	3	25	5	56	.185
“I feel comfortable prescribing nutritional interventions for disease management.”	2	18	1	11	1.000
“How much does your knowledge of integrative medicine/lifestyle medicine impact the type of care that you provide for your patients?” (Only “Moderately” or “Significantly”)	8	67	8	89	.733

a Absolute values correspond to the number of survey respondents who chose “Agree” or “Strongly Agree” on the questionnaire.

In open-ended qualitative feedback on the workshops, residents provided food-specific comments suggesting “hands-on introduction to ‘different’ foods such as bok choy or lesser known vegetables” and “a primer on basic foods and nutrition facts.” Content-specific comments included the suggestion for more “discussion around cost-effective ingredients” and that patients in future workshops “could benefit from being taught/learning the most effective ways to identify healthy, quality foods at the store.” Similar comments indicated that the workshop “should incorporate more of the challenges facing patients cooking in Baltimore,” including “cost of food, as it's difficult to imagine how patients will be able to afford some ingredients.” Skills-based comments suggested that future workshops should include training on the “structure of effective community cooking classes” and include “recipes without need for appliances.” Common themes were the importance of providing basic nutritional knowledge, of adapting materials to local culture and resources, and of considering the socioeconomic status of many patients from inner-city Baltimore.

## Discussion

Our results demonstrated feasibility in implementing a culinary medicine pilot within a medical residency program that utilizes a community partnership. In regard to our first educational objective, to increase culinary skills and home-cooking behaviors of participants, we observed a statistically significant increase in the number of residents reporting confidence in cooking ability from our first to last workshop. We did not, however, note an increase in home-cooking behaviors. Regarding our second educational objective, to discuss practical culinary skills and nutritional counseling relevant to patients in an underserved setting, we created a time for reflection after cooking in the community kitchen and collected qualitative feedback on our survey in which residents emphasized the importance of tailoring content of workshops to the majority low-income community members. In regard to our third educational objective, to demonstrate culinary skills and nutritional counseling in a community kitchen, all participants were assigned to work in small groups and achieved their assigned task in the recipe. We did not note, however, a higher level of confidence in delivering patient nutritional counseling based on resident survey responses. According to the framing theory, the first two stages of the learning cycle—concrete experience and reflective observation—were positively impacted by this interactive nutrition curriculum, which supports the outcomes seen. We believe that the latter two stages of the framing theory—abstract conceptualization and active experimentation—are logical next steps of this process. A formal debrief at the conclusion of each workshop can help address these stages of learning.

We learned that recipes should be affordable. Recipes should also be popular and culturally relevant for the intended community, with the inclusion of healthy substitutions, and recipes selected should be appropriate for needed appliances relative to the resources that community members have. These factors were considered based on feedback from residents and community members who were also invited to participate in these workshops. Curriculum development can and should occur with the assistance of community members, and their input on topics for education and recipes needs to be included. Although having a physician-chef involved was helpful in leading this pilot, it may not be necessary for future programs, and including a nutritionist or dietitian may be worthwhile in promoting interprofessional education.^15^ The use of PowerPoint should be limited to the initial workshops, and hands-on learning should increasingly be emphasized with time.

We did experience challenges in implementing these workshops. First, we needed to find time within the already densely packed residency curriculum. By integrating nutritional and community health educational milestones, we were able to create a robust 12-hour training within the residency program to meet our learning objectives. Given the time constraints in resident education, other programs may want to consider similar integration of milestones to devote one, two, or three culinary workshops within their curriculum. Tailoring the workshops to residents with varying levels of cooking expertise was one challenge that we encountered. We would suggest that future programs ask for the participants’ level of comfort prior to the workshop and assign tasks accordingly, with simpler tasks being assigned to those with less experience. Another challenge was choosing healthy and affordable recipes that were amenable to different parts of each recipe being completed by different teams. It is helpful to consult with a chef or a nutritional educator for larger groups of participants. Finally, some participants were less engaged with the cooking. Typically, this was related to their comfort in the kitchen. Again, knowing this information in advance and pairing these participants with those who are more experienced can be helpful.

Limitations include the small sample size and deidentified resident responses. Although statistical analysis of our data showed positive trends among the residents sampled, we suggest caution in the interpretation of these data given that this was a formative pilot study. The total number of residents who participated and completed surveys decreased from the initial workshop to the final workshop, and to protect the privacy and anonymity of residents, we did not link baseline responses to postcurriculum responses. If residents who reported high self-efficacy in cooking ability after the first workshop dropped out at higher rates than those reporting low self-efficacy, we would observe a false increase in reported self-efficacy. A larger sample of residents, possibly pooled across multiple academic years of this intervention, could improve the power to detect statistically significant results. In addition, conducting a knowledge assessment before and after each workshop would also enhance the ability to evaluate resident skills and should be considered in future implementations. A skills assessment after the pilot workshops would provide a useful tool to evaluate interventional success and resident culinary teaching and nutritional educational abilities in the community. This should also be considered as a next step.

Although self-assessment data (formative) did not include measurable data and serve as a limitation to our pilot analysis, we see this as an area of focus for future research. There was slight attrition in the resident sample primarily due to a small number of residents who missed the final workshop and/or were unable to complete the postworkshop questionnaire for the final workshop. Making efforts to schedule workshops at a convenient time for all residents and ensuring full integration into the residency curriculum as a required activity during a weekday (as opposed to a weekend day, which is when our workshops were held) would be suggestions to limit attrition in future studies. Although only three recipes are provided in the current pilot, facilitators are encouraged to develop additional recipes that are simple yet tailored to the community being served, ideally with input from residents regarding what may be most effective.

Statistically significant changes were not seen in a majority of secondary outcomes in our study, especially as related to self-efficacy. It is possible that our small sample size limited our efforts to distinguish changes from the first workshop to the final workshop. It is also possible that this workshop provides foundational learning in nutritional counseling; however, spiraling the curriculum in other settings is necessary to help reinforce and apply it to clinical care of patients.

The formative function of this pilot study provides valuable information to inform and shape our future curriculum efforts. In future scaled-up iterations of this program, we recommend the use of additional tools of evaluation or indicators to measure the impact of the intervention on behaviors of community members who participate in activities. We also recommend more efforts to perform robust qualitative and formative assessment, such as focus groups and participant one-on-one interviews. Conducting a formal qualitative analysis utilizing formative data from these sessions is suggested as a next step in the research process as well. Study facilitators may also consider adding items to the survey tool that inquire about family status (living alone or with a partner and/or children) to see whether this might influence the frequency of home-cooked meals and personal cooking competency as reported by residents and/or survey participants. The literature suggests a variety of additional indicators to evaluate cooking behavior and confidence, including reported fruit and vegetable consumption, and cooking frequency.^16^ More robust evaluation would require more extensive resources. Larger and more robust studies should follow to build on the initial strength of our pilot findings. Conducting a formal validation of the survey instrument would also provide additional quality to research findings and should be considered in future studies.

Our pilot highlights the possibility of resident culinary education to improve resident cooking practices. Clinicians who practice healthy personal behavior are more likely to counsel patients on these preventive behaviors.^17^ Responding more positively to being a competent cook may be a first step in prescribing nutritional interventions to patients in the future. In addition, implementing culinary education workshops through community-academic partnerships with institutions such as a local church or school may provide a unique opportunity for residents to gain knowledge of cooking challenges that patients and community members face, which may further enhance the effectiveness of such interventions.^18^

## Appendices

A. Facilitator Guide.docx
B. Workshop 1 Presentation.pptx
C. Workshop 2 Presentation.pptx
D. Workshop 3 Presentation.pptx
E. Tofu Lettuce Cups Recipe.pdf
F. Kale Pesto Recipe.pdf
G. Cold Asian Noodles Recipe.pdf
H. Postworkshop Survey.docx All appendices are peer reviewed as integral parts of the Original Publication.
