# Supplementary material for: Community Culinary Workshops as a Nutrition Curriculum in a Preventive Medicine Residency Program
Source: MedEdPORTAL. 2019 Dec 13;15:10859. doi: 10.15766/mep_2374-8265.10859 (PMC7010195; doi:10.15766/mep_2374-8265.10859)
Supplement: Supplementary file 1 — A. Facilitator Guide.docx B. Workshop 1 Presentation.pptx C. Workshop 2 Presentation.pptx D. Workshop 3 Presentation.pptx E. Tofu Lettuce Cups Recipe.pdf F. Kale Pesto Recipe.pdf G. Cold Asian Noodles Recipe.pdf H. Postworkshop Survey.docx [file mep-15-10859-s001.zip › A. Facilitator Guide.docx]

**Facilitator Guide**

Venue: Large community kitchen

Facilitators: 3 faculty members

**Session layout**

| **Activity** | **Time** |
| --- | --- |
| Introductions, food preferences/allergies, get to know audience, describe objectives | 15 minutes |
| Core didactics and facilitated discussion | 45 minutes |
| Orient to kitchen, assign work stations | 15 minutes |
| Make recipe | 45 minutes |
| Eat, debrief and reflect on relevance to target community | 60 minutes |
| Clean up | 30 minutes |
| Wrap up, plans for future sessions, questions, complete survey | 15 minutes |

Practical instructions:

1. Introductions:

Be certain to get a sense of level of experience in the kitchen, food preferences and any allergies at the start of the session. This helps in assigning roles in the kitchen. If possible, ask for preregistration of attendees with these questions. This helps to assign and set up workstations in the kitchen beforehand.

1. Core didactics and facilitated discussion:

This takes 45 minutes and may include a slide presentation as a visual aid (though not required). Take time to pose questions to the audience and encourage questions. Make this as interactive as possible and consider using brief demonstrations of simple cooking skills to introduce certain concepts to learners before they enter the kitchen.

1. Orient to kitchen, assign work stations

This is a critical component. Have the learners work in groups of 3-4. Have recipes available at each workstation and review key steps of each part of the recipe for all learners. Review importance of communication in kitchen, safety, and hygiene.

1. Make recipe

All learners should be actively taking part in the creating the recipe.

1. Lead facilitator provides instructions for each of the workstations and gives a timeline to complete the tasks. She/he directs flow of the kitchen and keeps everyone on time.
2. Facilitators should provide short demonstrations at each of the workstations and provide help as needed

- Typical pitfall:
  - Some learners may be assigned to workstations in which they have some level of experience and other learners may be assigned to workstations in which they have no experience. It may be useful to have learners rotate during workshops so that learners can have experience with multiple parts of the recipe.

1. Debrief and reflect on relevance to target community
2. Allow for natural conversations regarding the recipe, how the food tastes, and ways to improve in the future. Take 10 minutes at the end of the workshop to ask questions perhaps in roundtable format, to residents and/or other workshop participants.
3. The debrief adds to the explicit experience. Consider asking the following questions:

- What challenges were faced when working in the kitchen?
- How difficult was it to make the recipe?
- What resources were lacking and how was this overcome?
- How would the community members handle making the food at home?
- Would community members enjoy the recipe and the taste of the food?

1. Record comments from debrief session on paper and/or consider using audio to record conversation in order to transcribe later for the identification of thematic content which may be used later for formal qualitative analysis.
2. Clean up

It is important to emphasize the need to clean up the kitchen as part of the natural process of cooking. Learners should be encouraged to clean up throughout the cooking workshop and not wait until the end of the workshop to do all clean-up. However, time should be allotted during the workshop to finalize the clean-up process.

1. Wrap up

If the debrief is done as described, there should be very few questions; however, leaving a few minutes for lingering questions and survey completion as well as planning for the next workshop is helpful.

1. Facilitation pearls
   1. It is important to know who the learners are, their comfort level in the kitchen, their food preferences and any allergies, and assign them to groups/tasks accordingly.
   2. Any time there is a person that is more experienced in the kitchen available, be sure to use that person’s experience. It is helpful to have these natural leaders in the kitchen be assigned to different workstations.
   3. Choose recipes that are affordable, healthy, and simple to make. Think ahead regarding feasibility of breaking recipes apart into stations and then bringing ingredients and teams together when needed.
   4. There are some people who do not feel comfortable in the kitchen. It is critical to encourage them. The more involved that they get, the better the learning. Often keeping their role simple helps as well as pairing them with comfortable leaders in the kitchen.

Materials: Microsoft PowerPoint slides and recipes
