# Supplementary material for: Community Culinary Workshops as a Nutrition Curriculum in a Preventive Medicine Residency Program
Source: MedEdPORTAL. 2019 Dec 13;15:10859. doi: 10.15766/mep_2374-8265.10859 (PMC7010195; doi:10.15766/mep_2374-8265.10859)
Supplement: Supplementary file 1 — A. Facilitator Guide.docx B. Workshop 1 Presentation.pptx C. Workshop 2 Presentation.pptx D. Workshop 3 Presentation.pptx E. Tofu Lettuce Cups Recipe.pdf F. Kale Pesto Recipe.pdf G. Cold Asian Noodles Recipe.pdf H. Postworkshop Survey.docx [file mep-15-10859-s001.zip › B. Workshop 1 Presentation.pptx]

## Slide 1
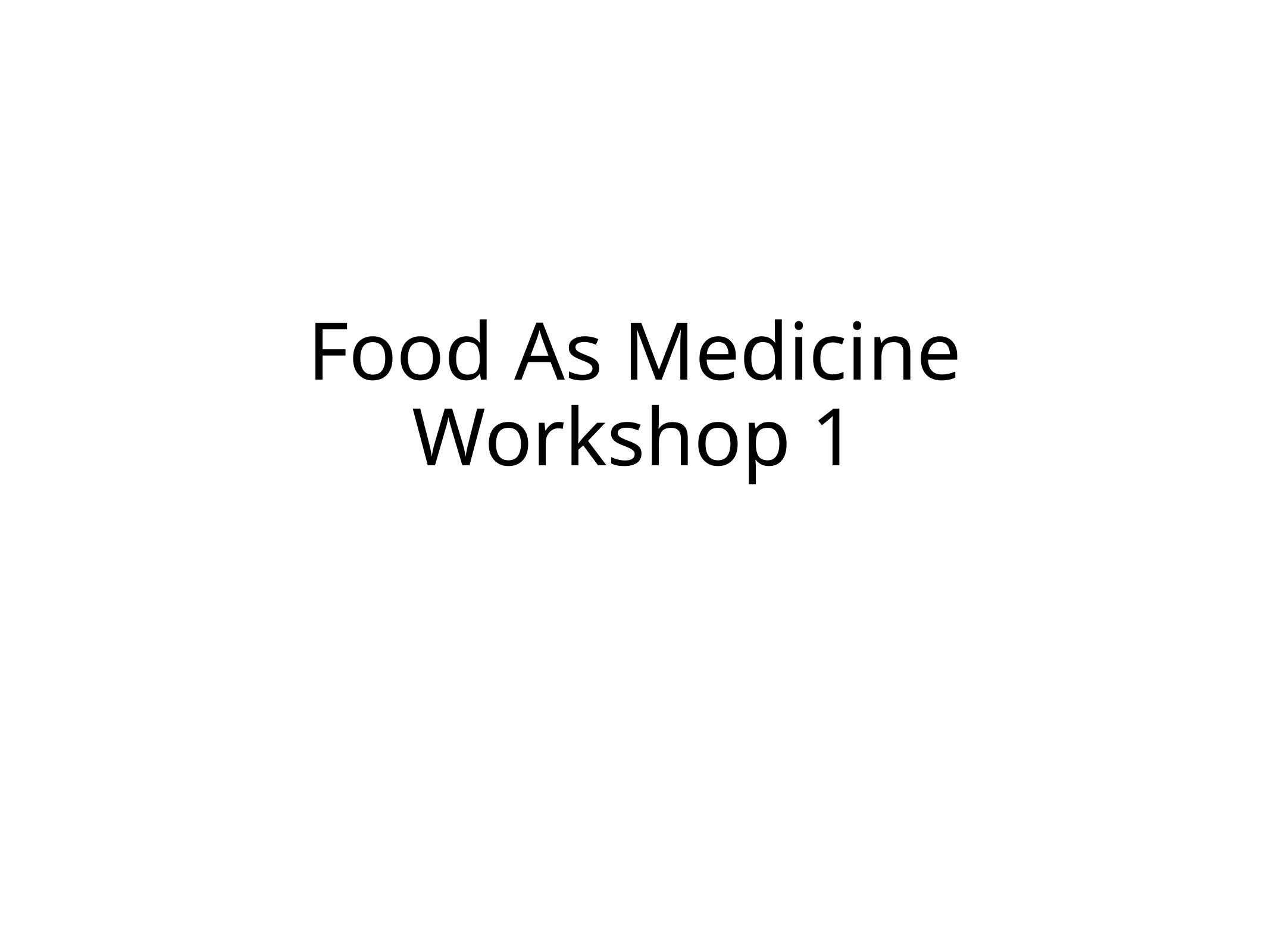

# Food As Medicine Workshop 1

## Slide 2
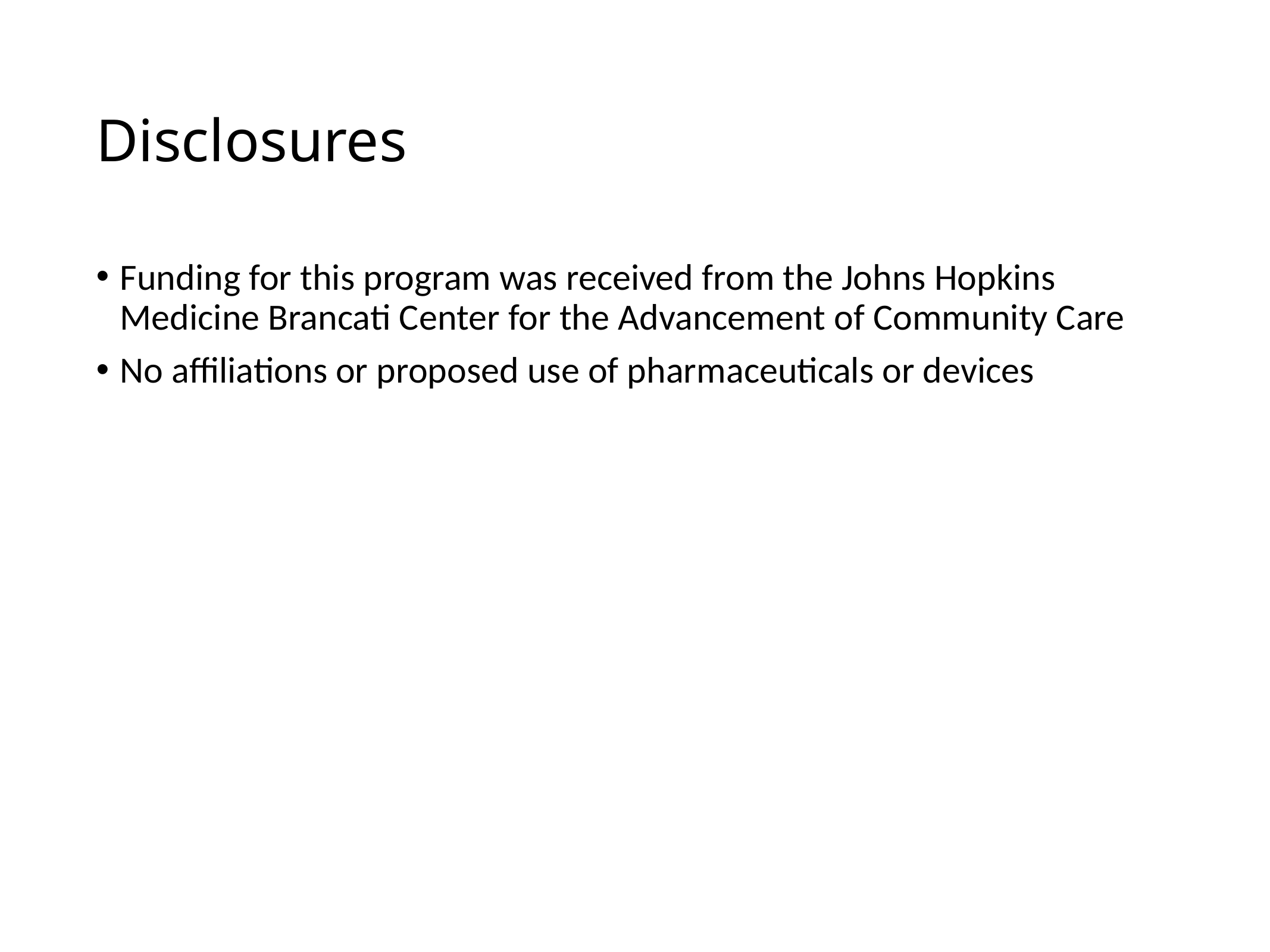

# Disclosures
Funding for this program was received from the Johns Hopkins Medicine Brancati Center for the Advancement of Community Care
No affiliations or proposed use of pharmaceuticals or devices

## Slide 3
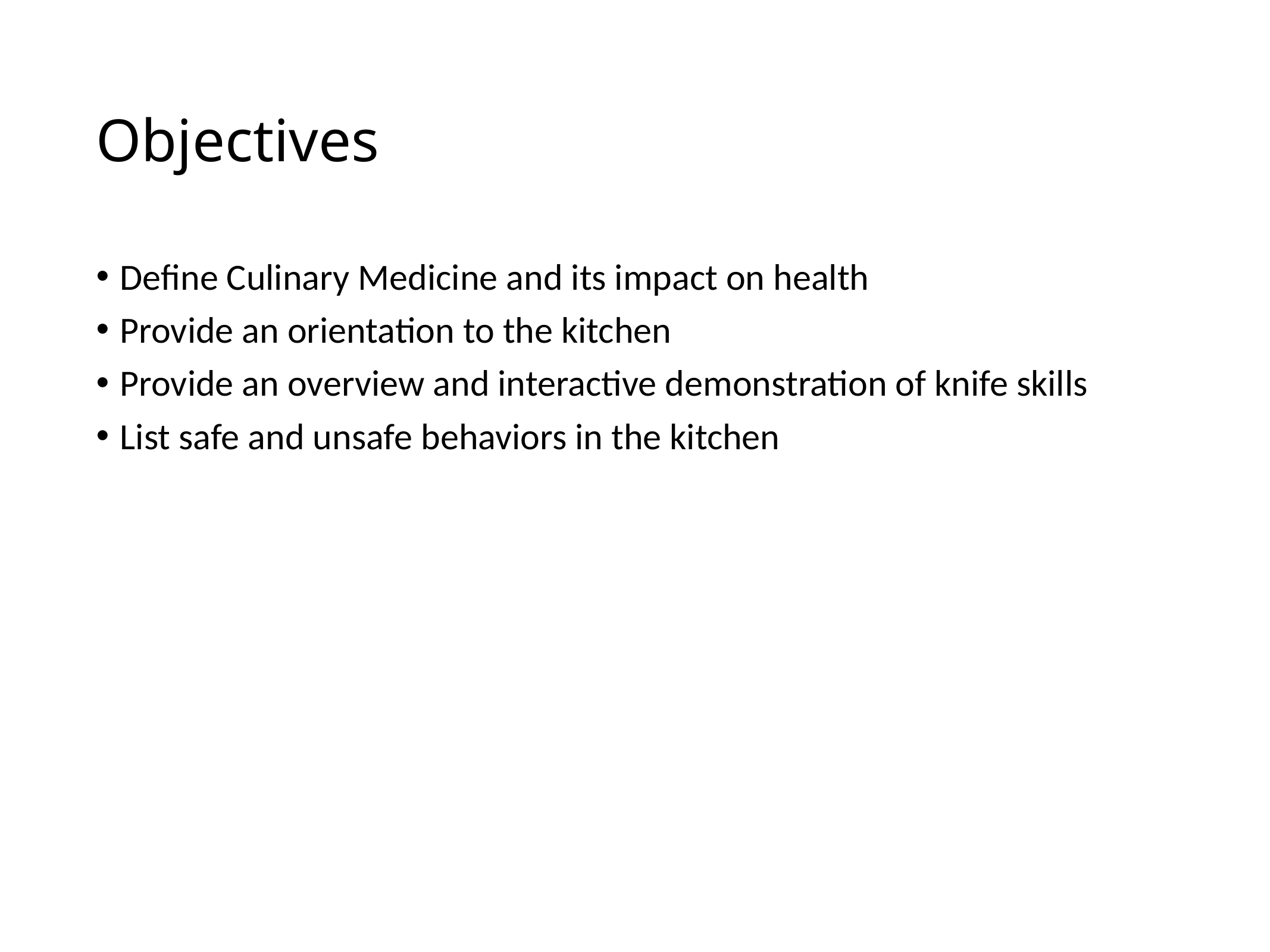

# Objectives
Define Culinary Medicine and its impact on health
Provide an orientation to the kitchen
Provide an overview and interactive demonstration of knife skills
List safe and unsafe behaviors in the kitchen

## Slide 4
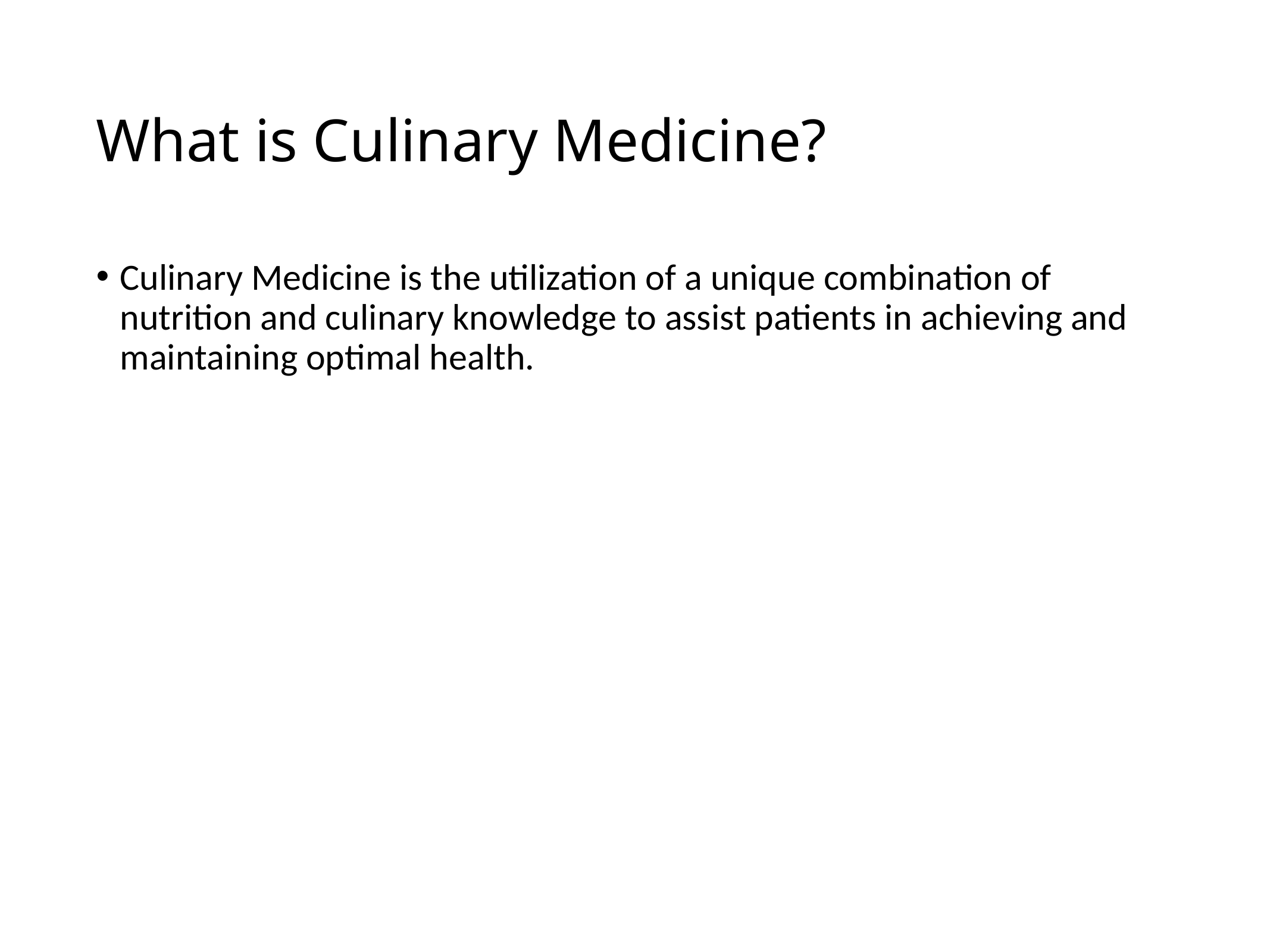

# What is Culinary Medicine?
Culinary Medicine is the utilization of a unique combination of nutrition and culinary knowledge to assist patients in achieving and maintaining optimal health.
La Puma, J. (2016). What is culinary medicine and what does it do?. Population health management, 19(1), 1-3.

## Slide 5
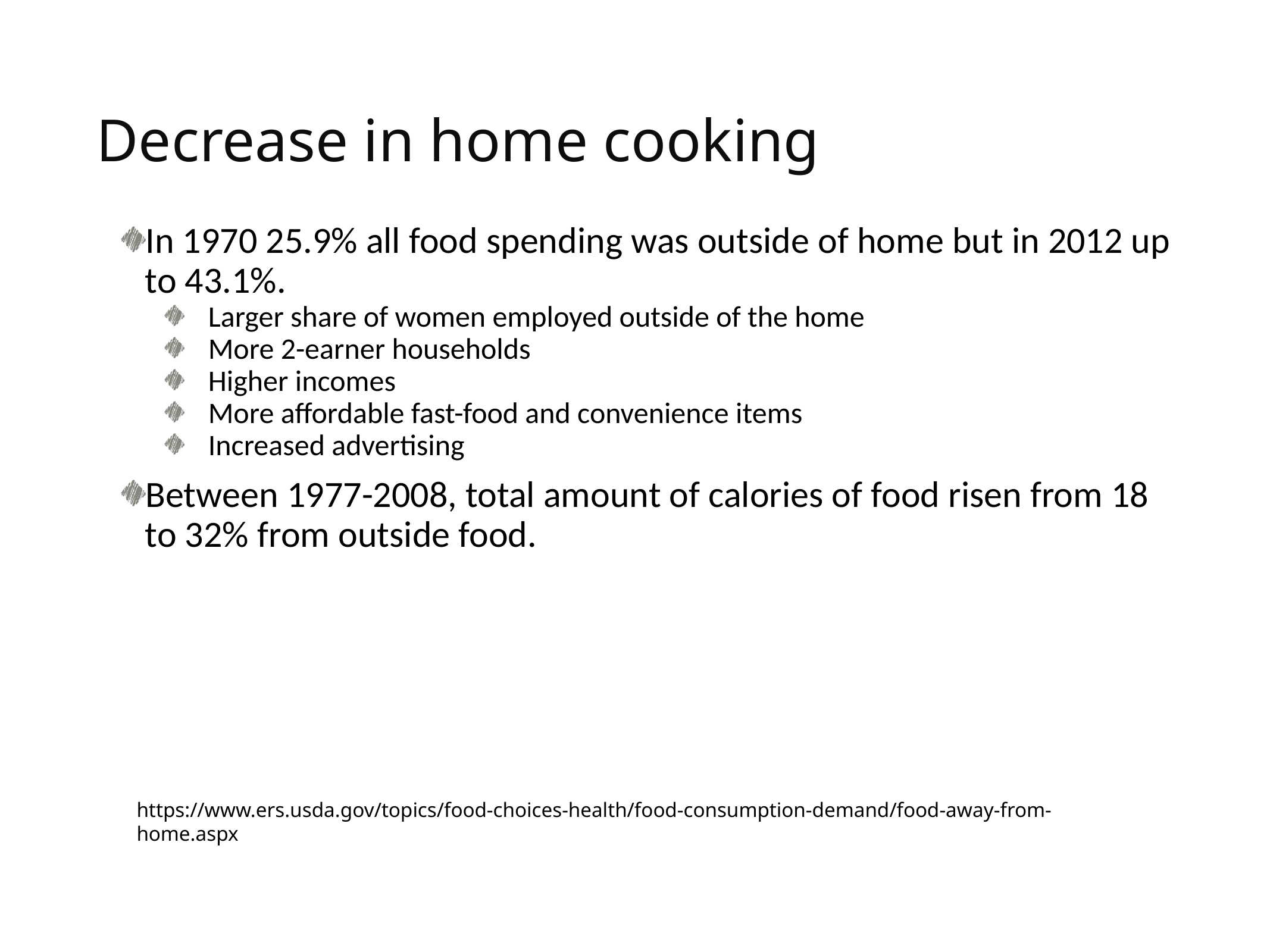

# Decrease in home cooking
In 1970 25.9% all food spending was outside of home but in 2012 up to 43.1%.
Larger share of women employed outside of the home
More 2-earner households
Higher incomes
More affordable fast-food and convenience items
Increased advertising
Between 1977-2008, total amount of calories of food risen from 18 to 32% from outside food.
https://www.ers.usda.gov/topics/food-choices-health/food-consumption-demand/food-away-from-home.aspx

## Slide 6
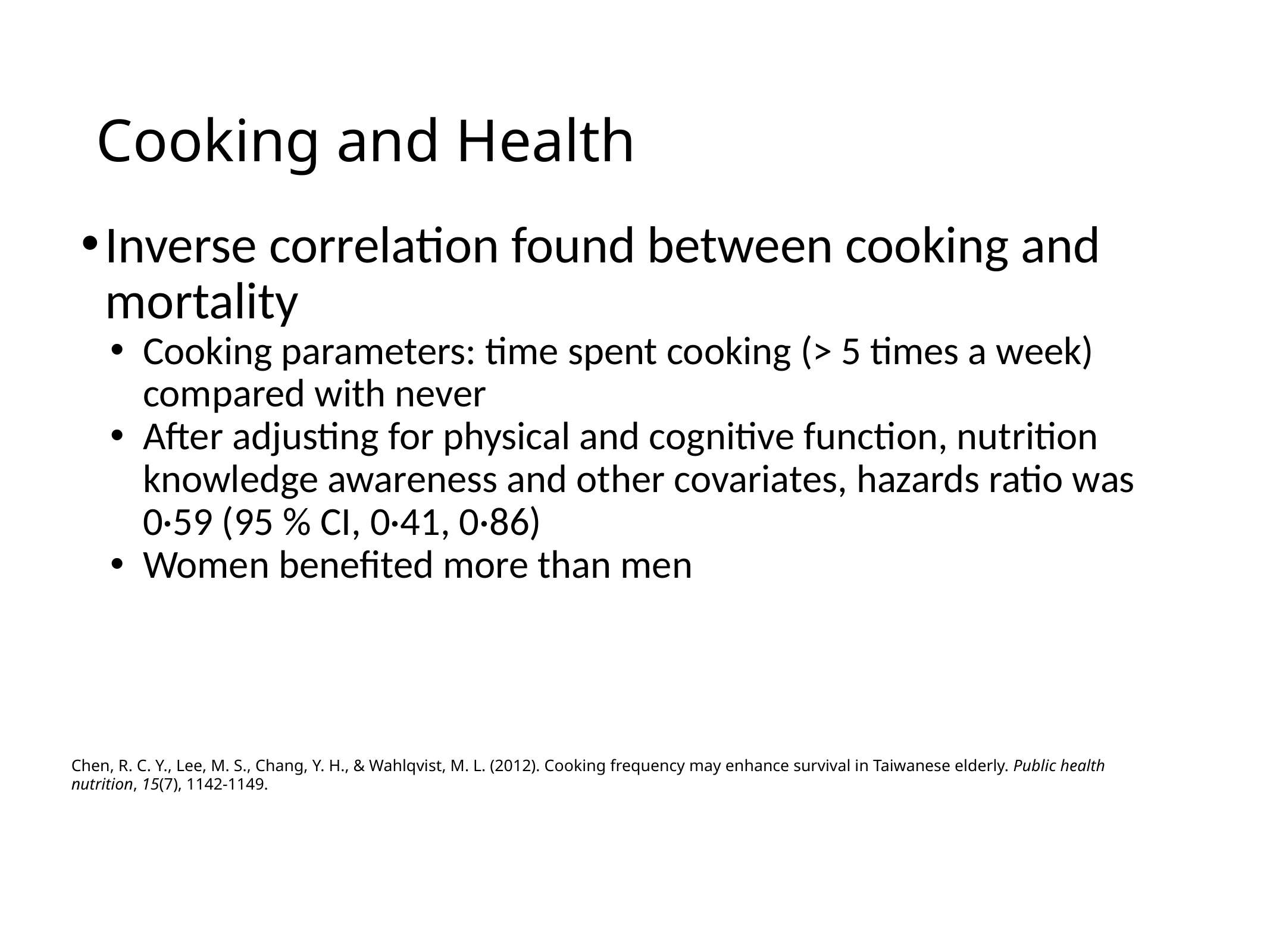

# Cooking and Health
Inverse correlation found between cooking and mortality
Cooking parameters: time spent cooking (> 5 times a week) compared with never
After adjusting for physical and cognitive function, nutrition knowledge awareness and other covariates, hazards ratio was 0·59 (95 % CI, 0·41, 0·86)
Women benefited more than men
Chen, R. C. Y., Lee, M. S., Chang, Y. H., & Wahlqvist, M. L. (2012). Cooking frequency may enhance survival in Taiwanese elderly. Public health nutrition, 15(7), 1142-1149.

## Slide 7
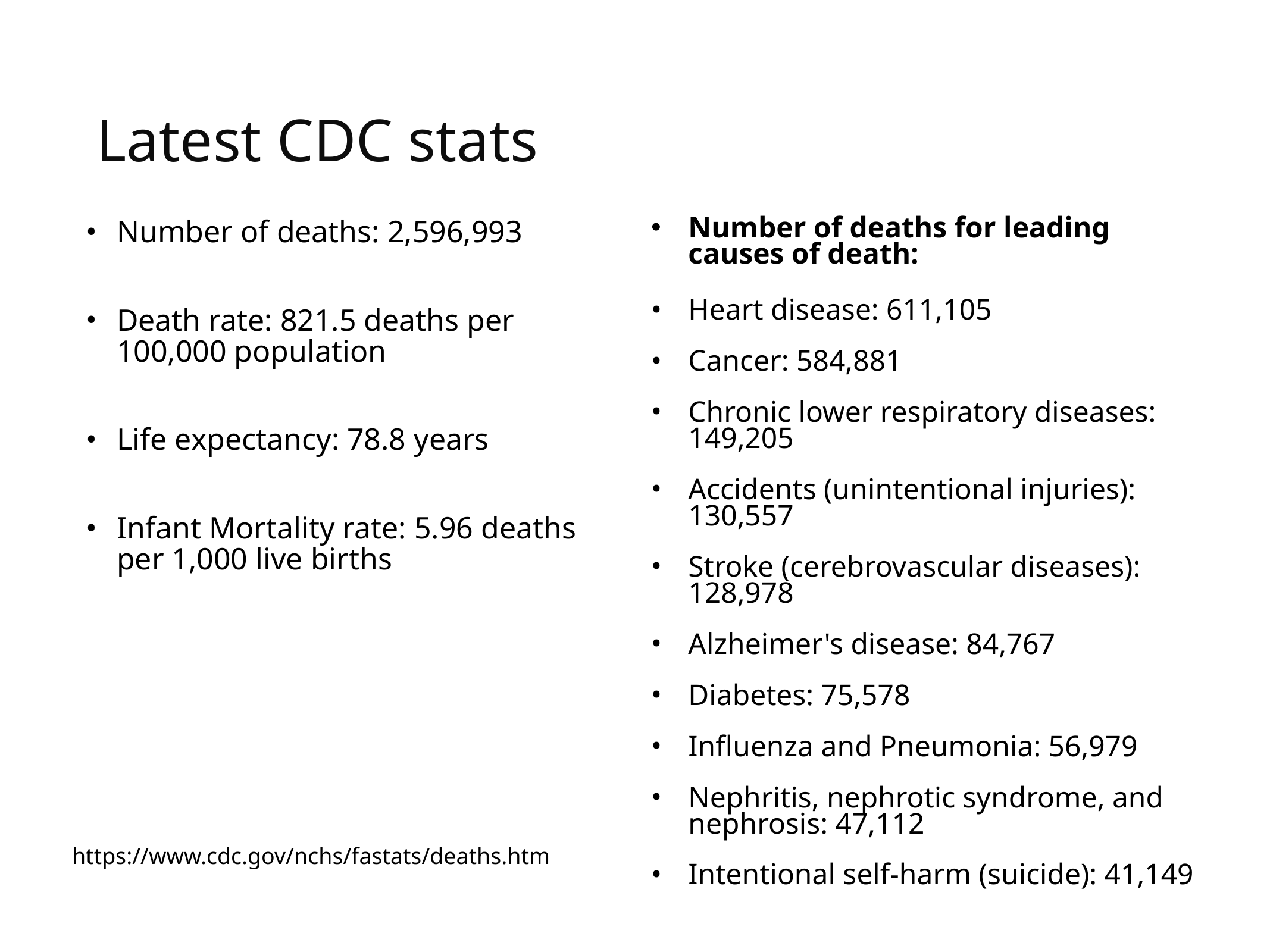

# Latest CDC stats
Number of deaths: 2,596,993
Death rate: 821.5 deaths per 100,000 population
Life expectancy: 78.8 years
Infant Mortality rate: 5.96 deaths per 1,000 live births
Number of deaths for leading causes of death:
Heart disease: 611,105
Cancer: 584,881
Chronic lower respiratory diseases: 149,205
Accidents (unintentional injuries): 130,557
Stroke (cerebrovascular diseases): 128,978
Alzheimer's disease: 84,767
Diabetes: 75,578
Influenza and Pneumonia: 56,979
Nephritis, nephrotic syndrome, and nephrosis: 47,112
Intentional self-harm (suicide): 41,149
https://www.cdc.gov/nchs/fastats/deaths.htm

## Slide 8
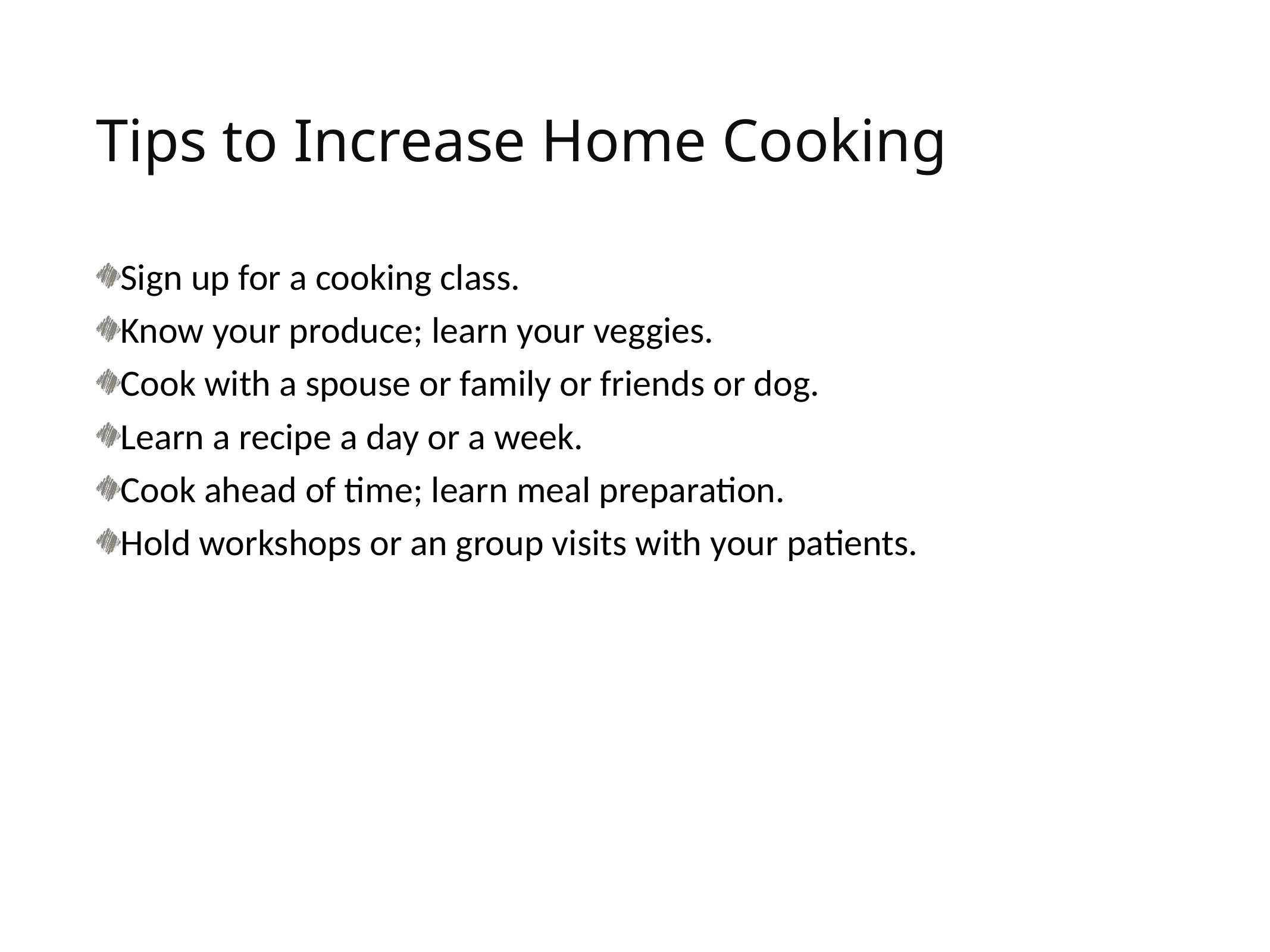

# Tips to Increase Home Cooking
Sign up for a cooking class.
Know your produce; learn your veggies.
Cook with a spouse or family or friends or dog.
Learn a recipe a day or a week.
Cook ahead of time; learn meal preparation.
Hold workshops or an group visits with your patients.

## Slide 9
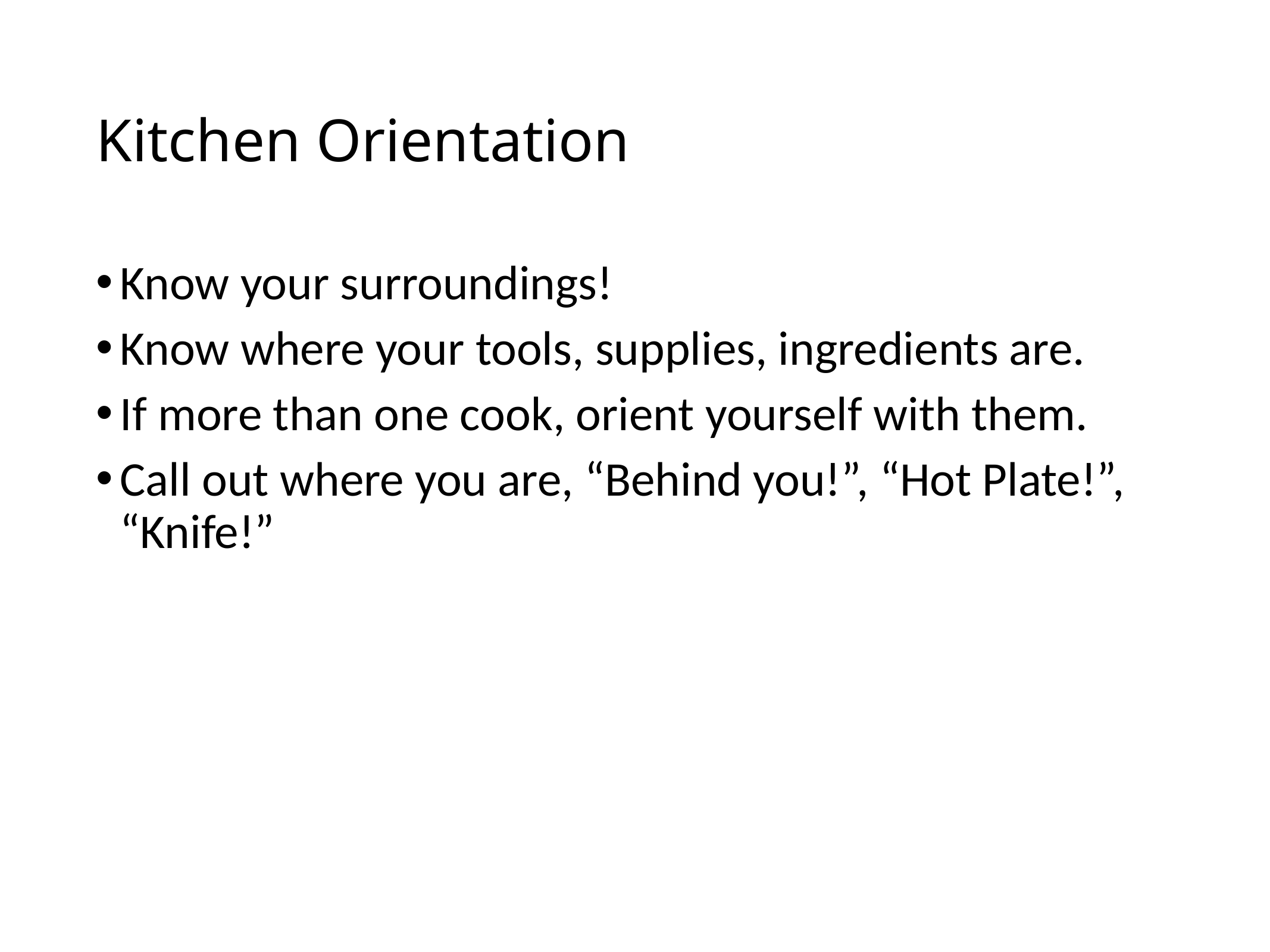

# Kitchen Orientation
Know your surroundings!
Know where your tools, supplies, ingredients are.
If more than one cook, orient yourself with them.
Call out where you are, “Behind you!”, “Hot Plate!”, “Knife!”

## Slide 10
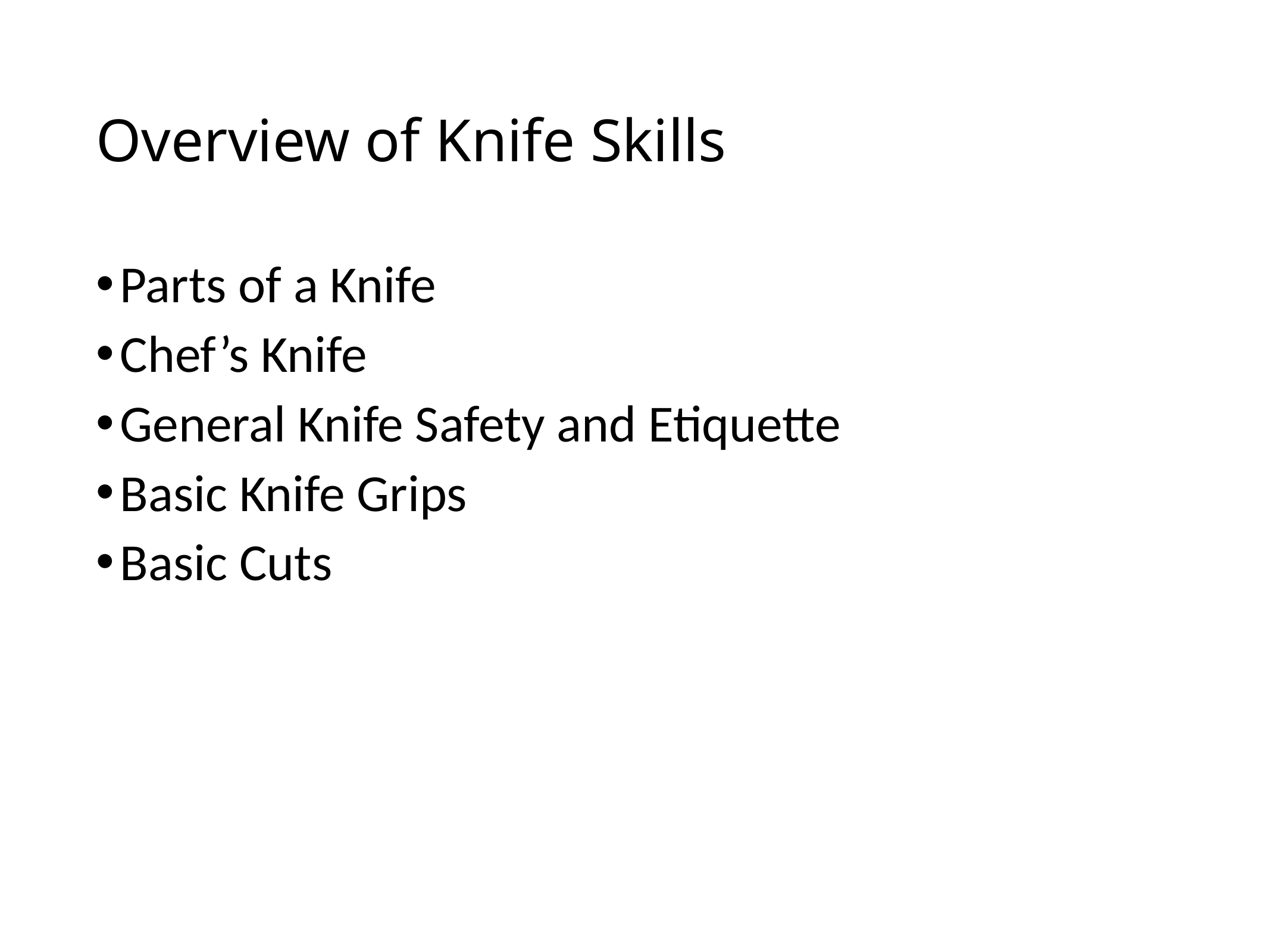

# Overview of Knife Skills
Parts of a Knife
Chef’s Knife
General Knife Safety and Etiquette
Basic Knife Grips
Basic Cuts

## Slide 11
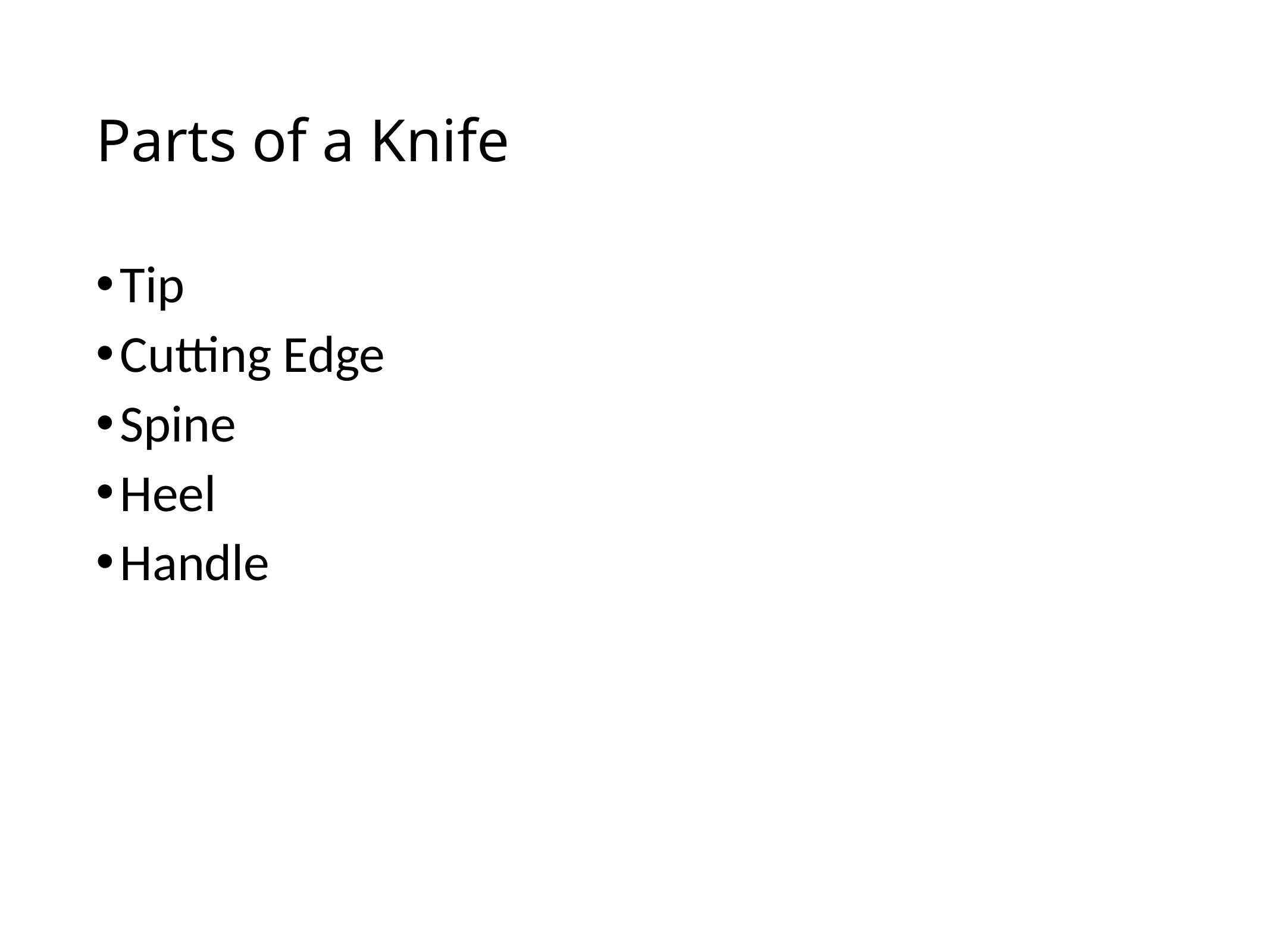

# Parts of a Knife
Tip
Cutting Edge
Spine
Heel
Handle

## Slide 12
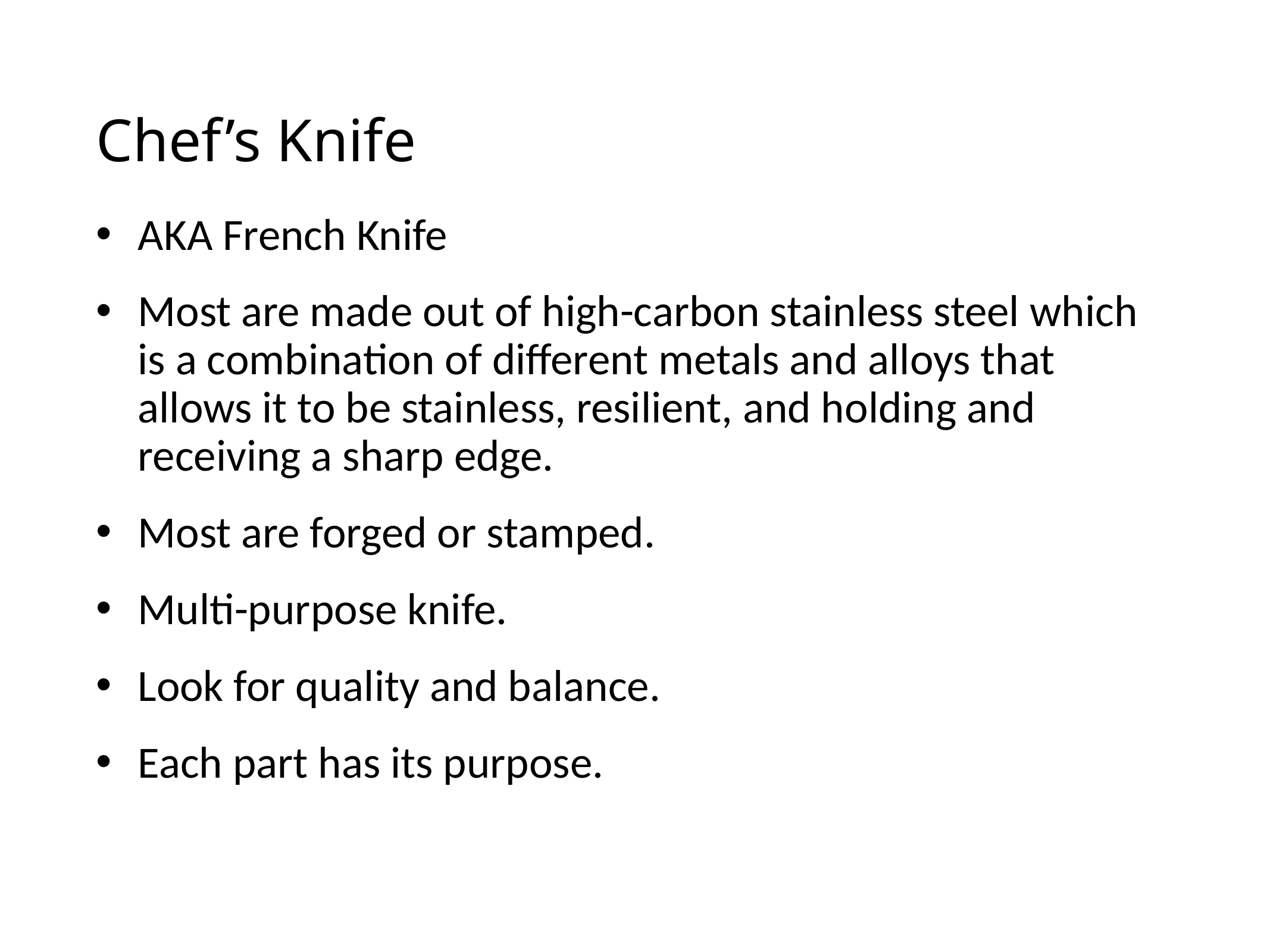

# Chef’s Knife
AKA French Knife
Most are made out of high-carbon stainless steel which is a combination of different metals and alloys that allows it to be stainless, resilient, and holding and receiving a sharp edge.
Most are forged or stamped.
Multi-purpose knife.
Look for quality and balance.
Each part has its purpose.

## Slide 13
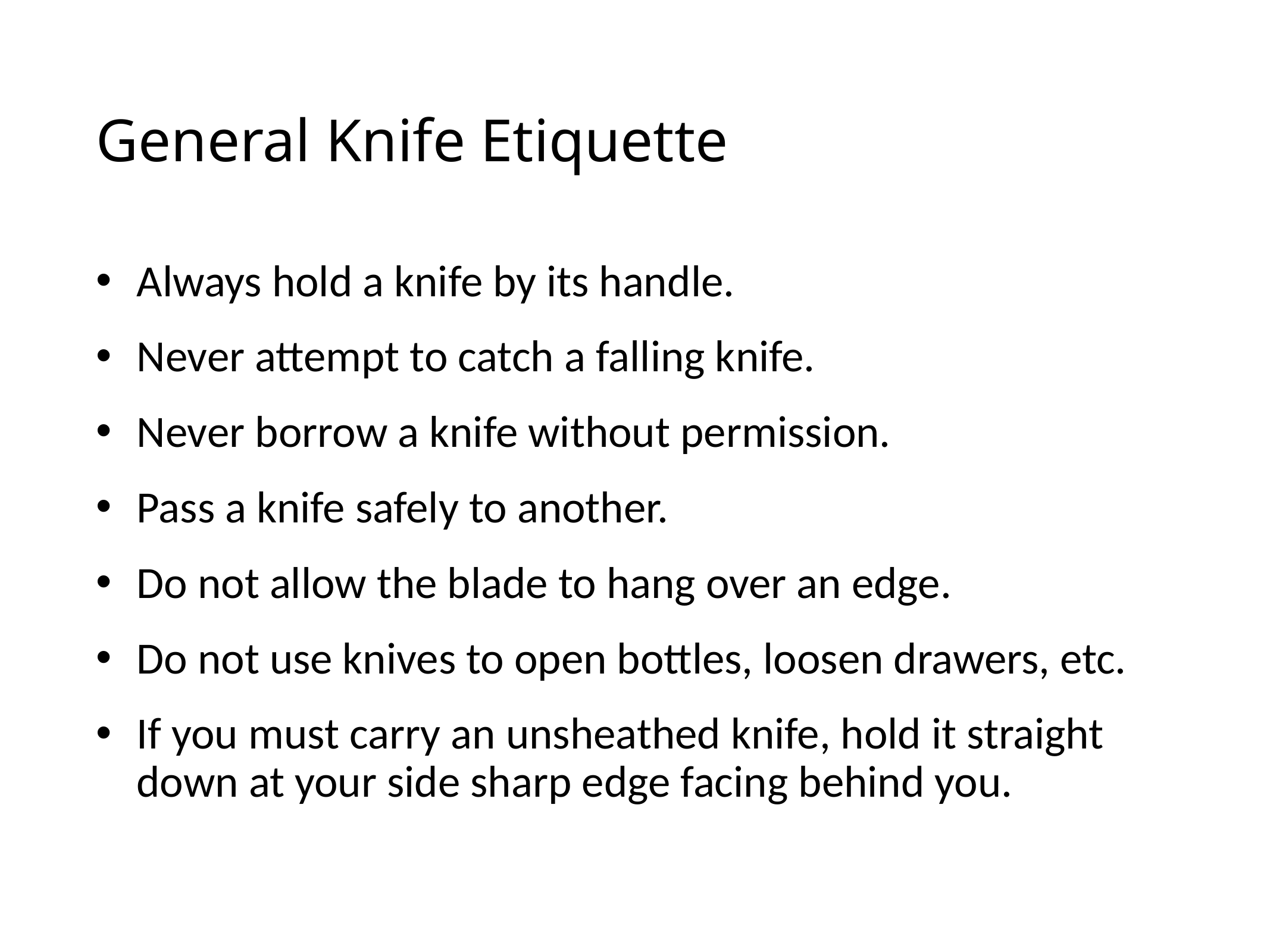

# General Knife Etiquette
Always hold a knife by its handle.
Never attempt to catch a falling knife.
Never borrow a knife without permission.
Pass a knife safely to another.
Do not allow the blade to hang over an edge.
Do not use knives to open bottles, loosen drawers, etc.
If you must carry an unsheathed knife, hold it straight down at your side sharp edge facing behind you.

## Slide 14
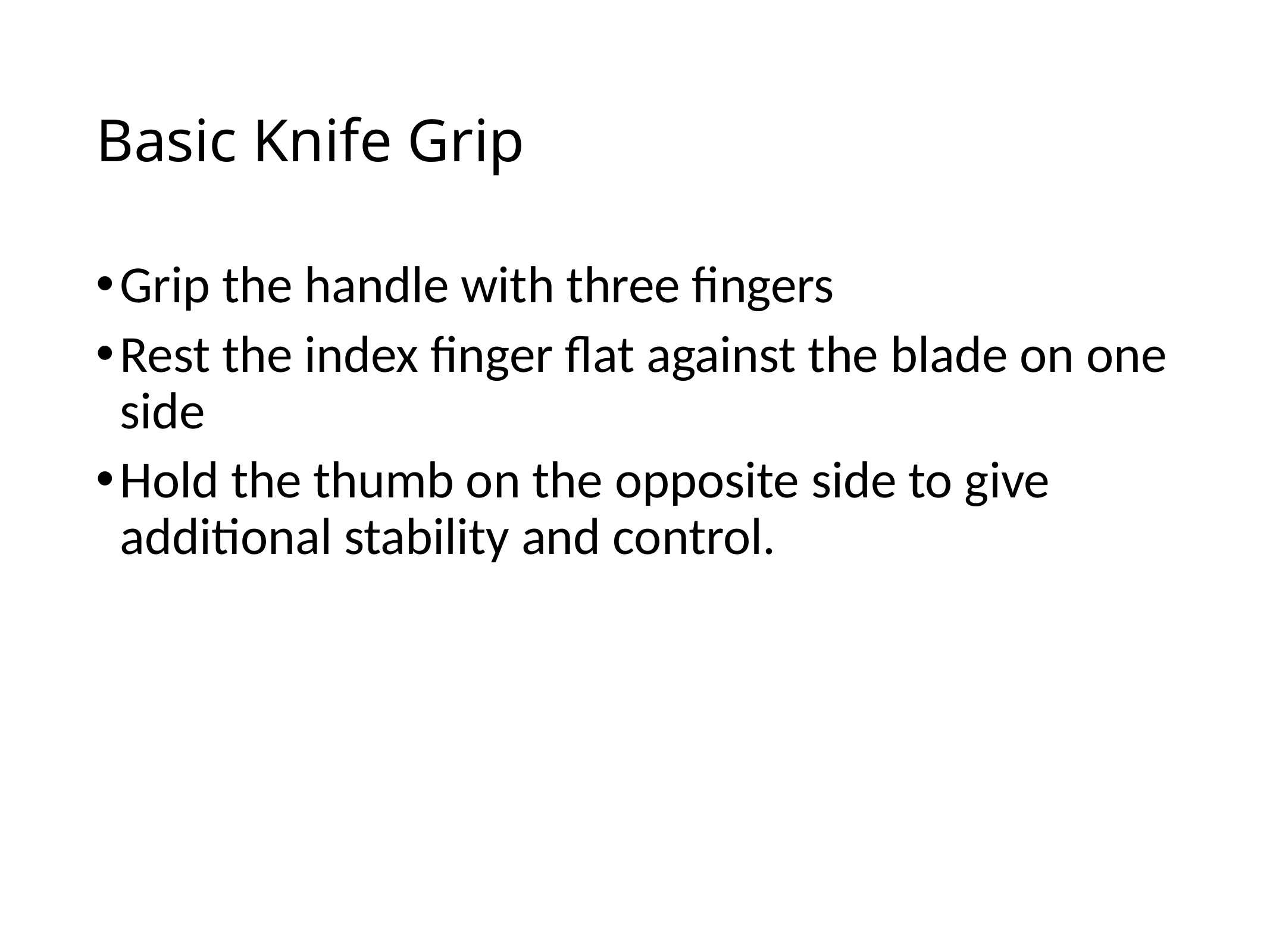

# Basic Knife Grip
Grip the handle with three fingers
Rest the index finger flat against the blade on one side
Hold the thumb on the opposite side to give additional stability and control.

## Slide 15
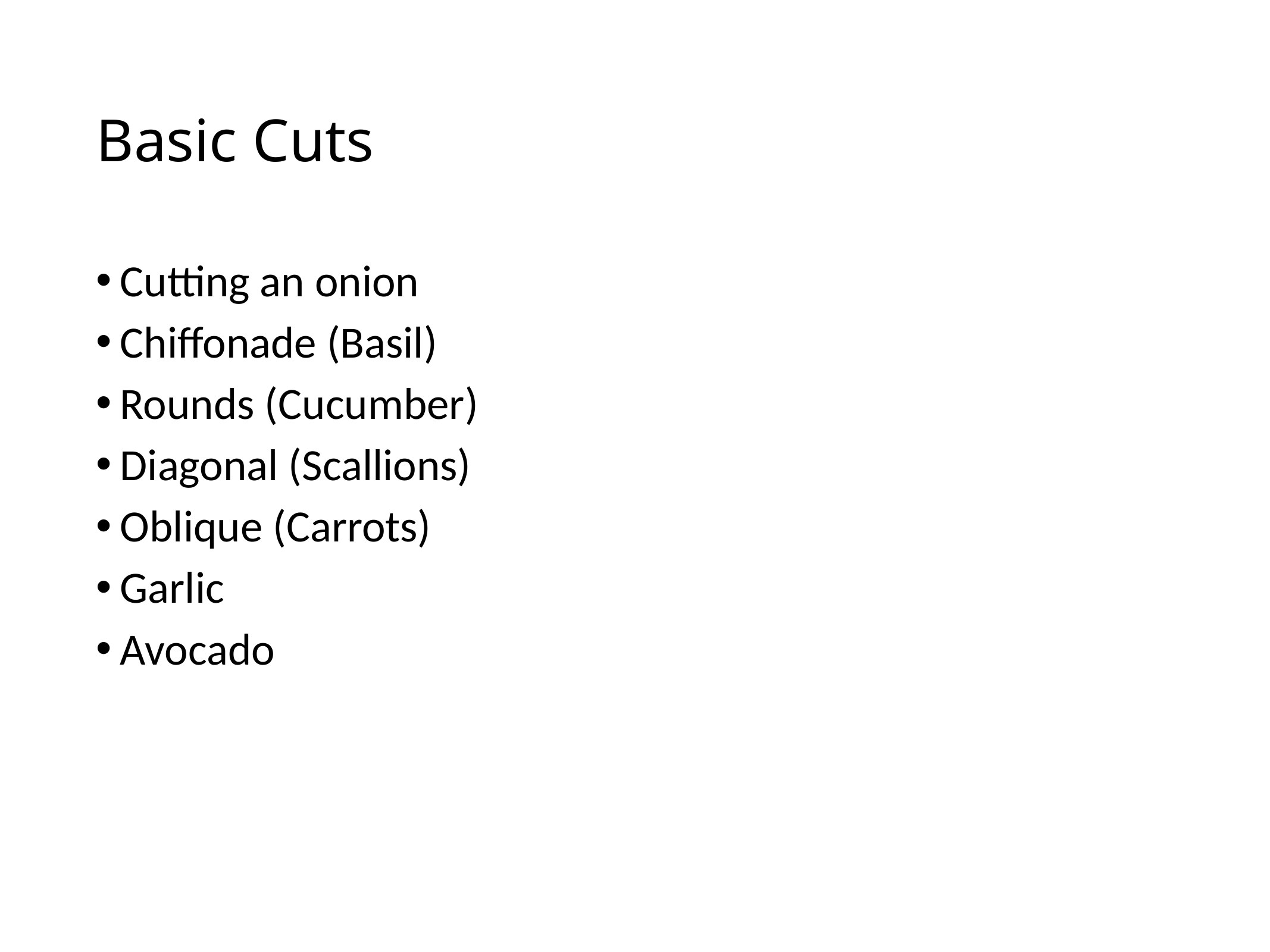

# Basic Cuts
Cutting an onion
Chiffonade (Basil)
Rounds (Cucumber)
Diagonal (Scallions)
Oblique (Carrots)
Garlic
Avocado

## Slide 16
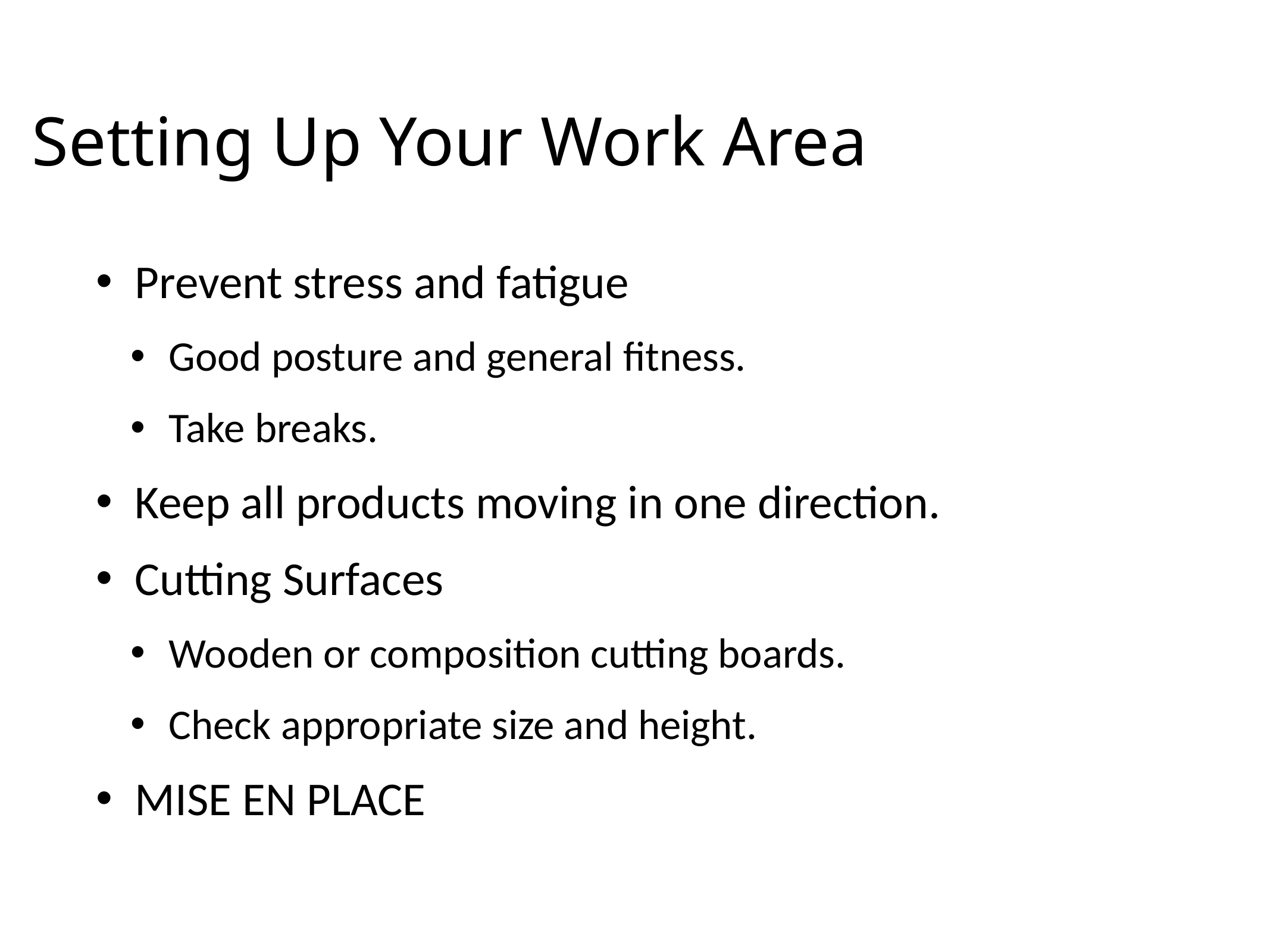

# Setting Up Your Work Area
Prevent stress and fatigue
Good posture and general fitness.
Take breaks.
Keep all products moving in one direction.
Cutting Surfaces
Wooden or composition cutting boards.
Check appropriate size and height.
MISE EN PLACE

## Slide 17
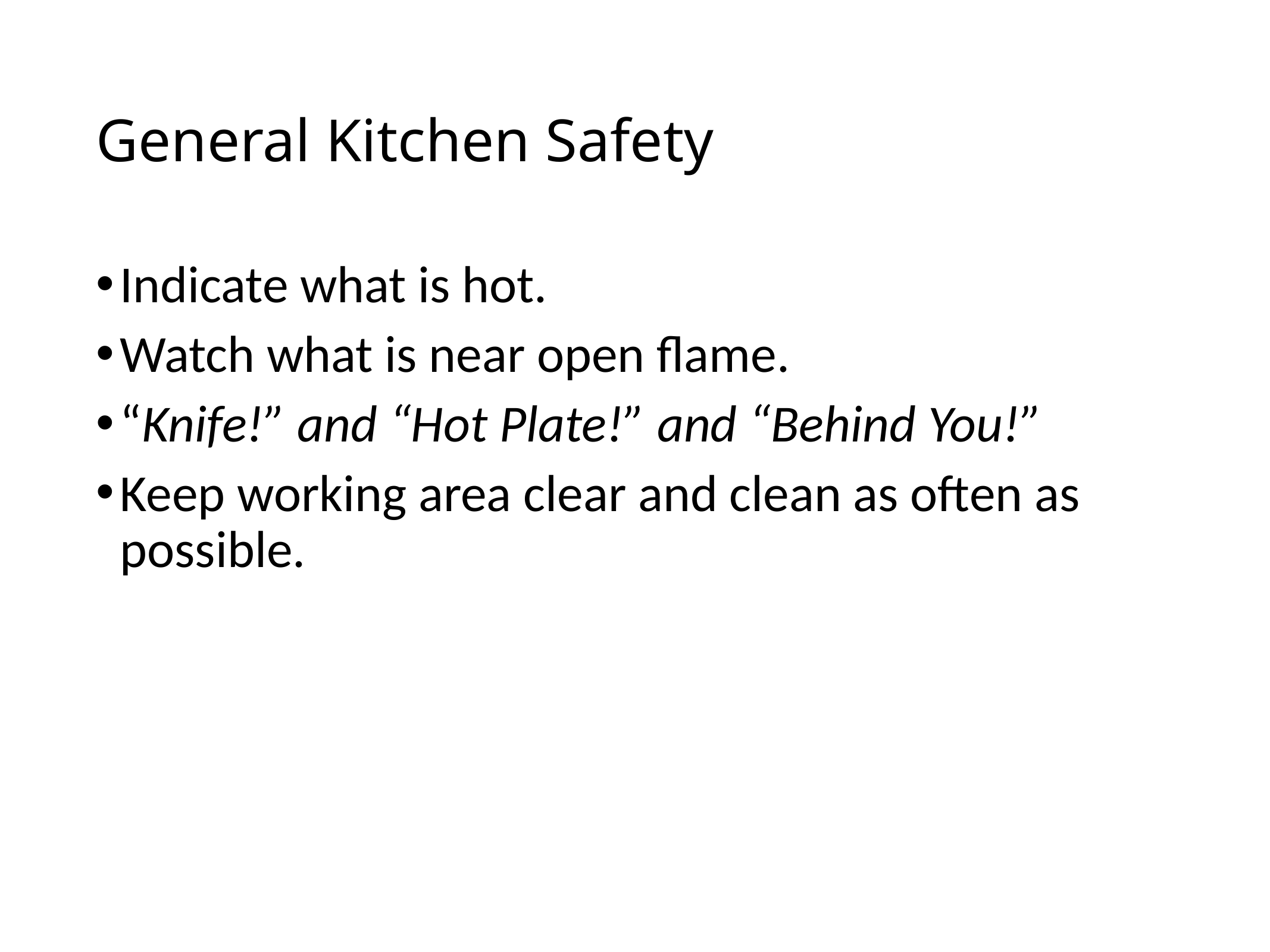

# General Kitchen Safety
Indicate what is hot.
Watch what is near open flame.
“Knife!” and “Hot Plate!” and “Behind You!”
Keep working area clear and clean as often as possible.

## Slide 18
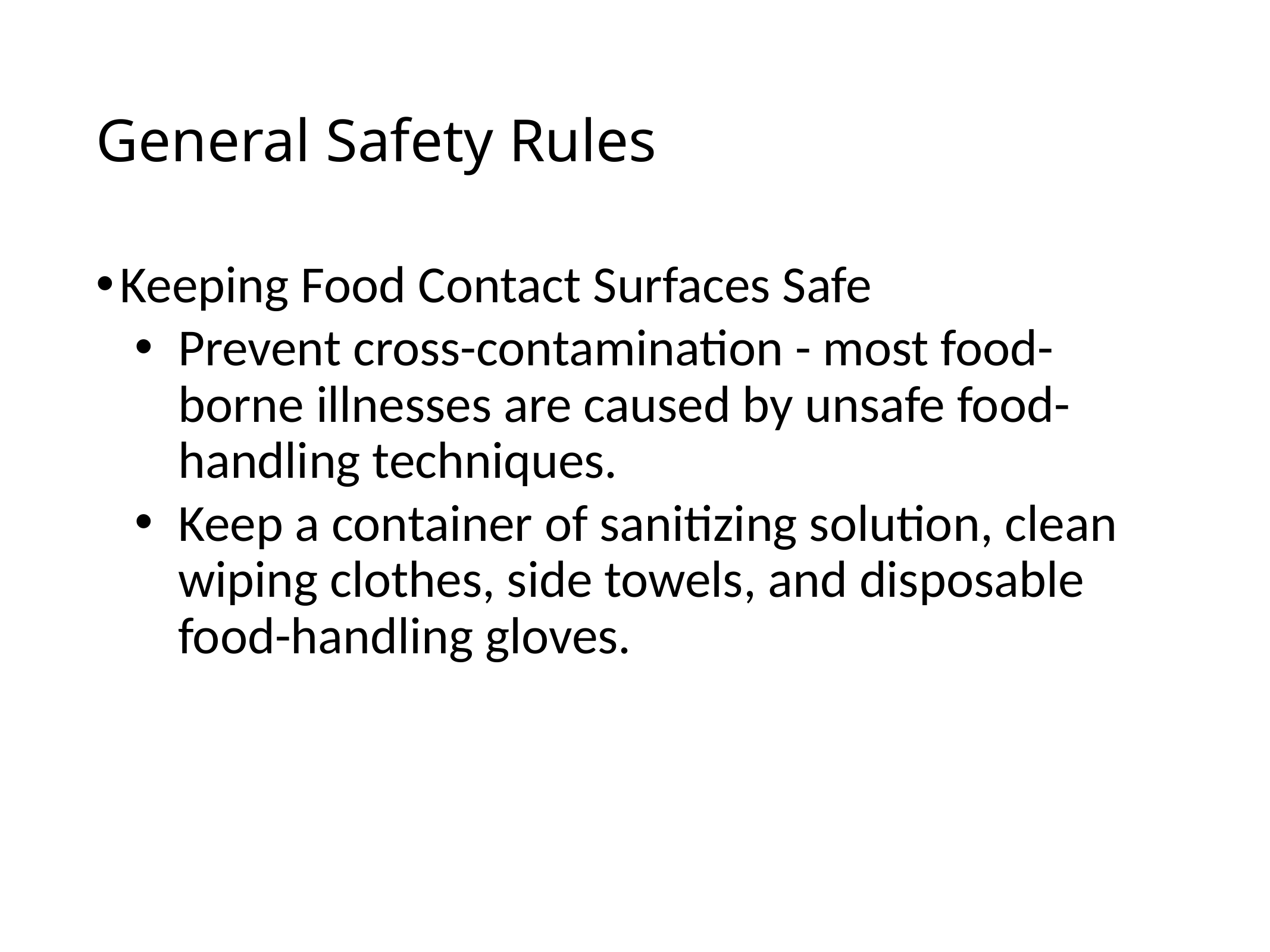

# General Safety Rules
Keeping Food Contact Surfaces Safe
Prevent cross-contamination - most food-borne illnesses are caused by unsafe food-handling techniques.
Keep a container of sanitizing solution, clean wiping clothes, side towels, and disposable food-handling gloves.

## Slide 19
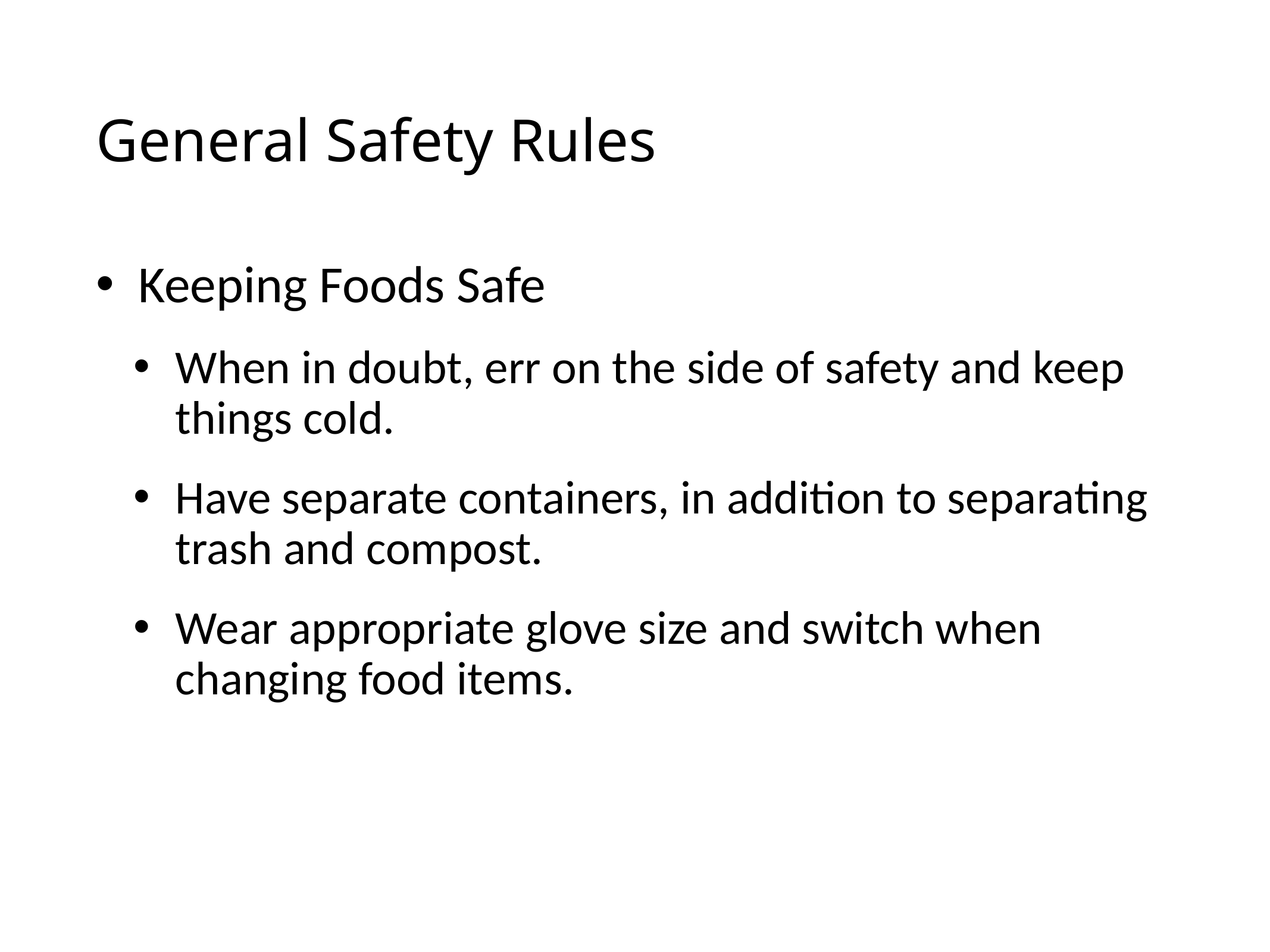

# General Safety Rules
Keeping Foods Safe
When in doubt, err on the side of safety and keep things cold.
Have separate containers, in addition to separating trash and compost.
Wear appropriate glove size and switch when changing food items.

## Slide 20
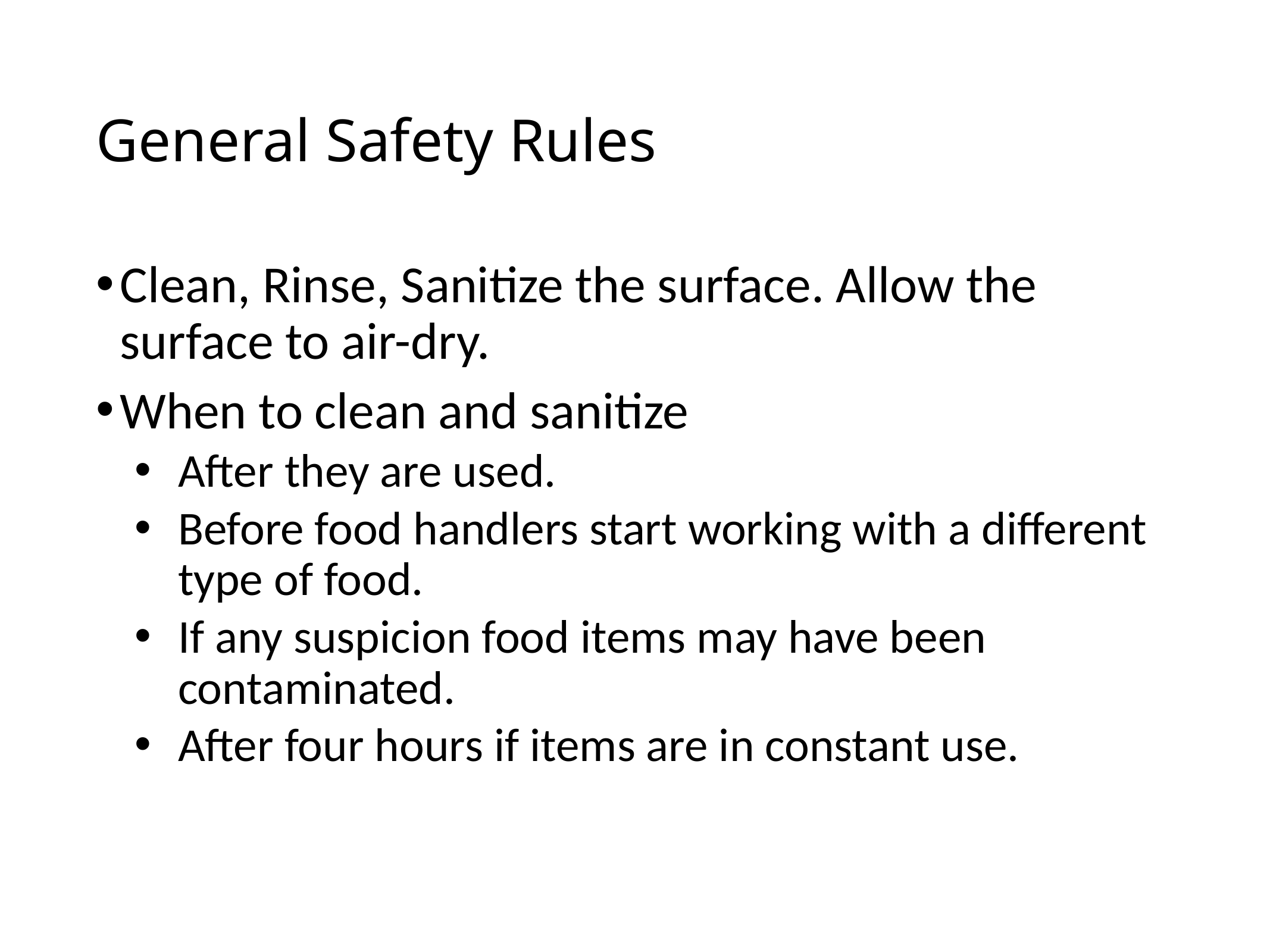

# General Safety Rules
Clean, Rinse, Sanitize the surface. Allow the surface to air-dry.
When to clean and sanitize
After they are used.
Before food handlers start working with a different type of food.
If any suspicion food items may have been contaminated.
After four hours if items are in constant use.

## Slide 21
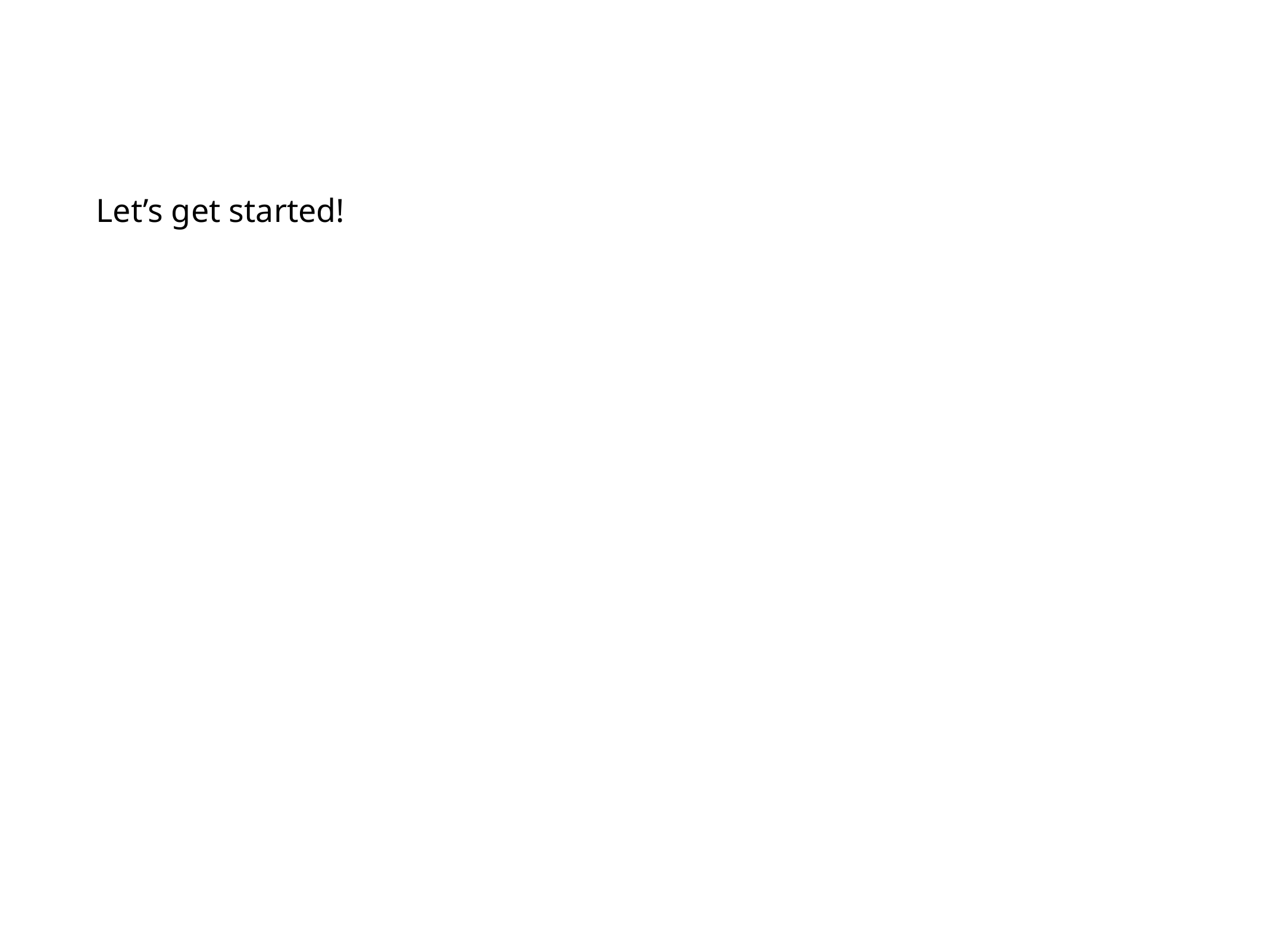

# Let’s get started!
