# Supplementary material for: Community Culinary Workshops as a Nutrition Curriculum in a Preventive Medicine Residency Program
Source: MedEdPORTAL. 2019 Dec 13;15:10859. doi: 10.15766/mep_2374-8265.10859 (PMC7010195; doi:10.15766/mep_2374-8265.10859)
Supplement: Supplementary file 1 — A. Facilitator Guide.docx B. Workshop 1 Presentation.pptx C. Workshop 2 Presentation.pptx D. Workshop 3 Presentation.pptx E. Tofu Lettuce Cups Recipe.pdf F. Kale Pesto Recipe.pdf G. Cold Asian Noodles Recipe.pdf H. Postworkshop Survey.docx [file mep-15-10859-s001.zip › C. Workshop 2 Presentation.pptx]

## Slide 1
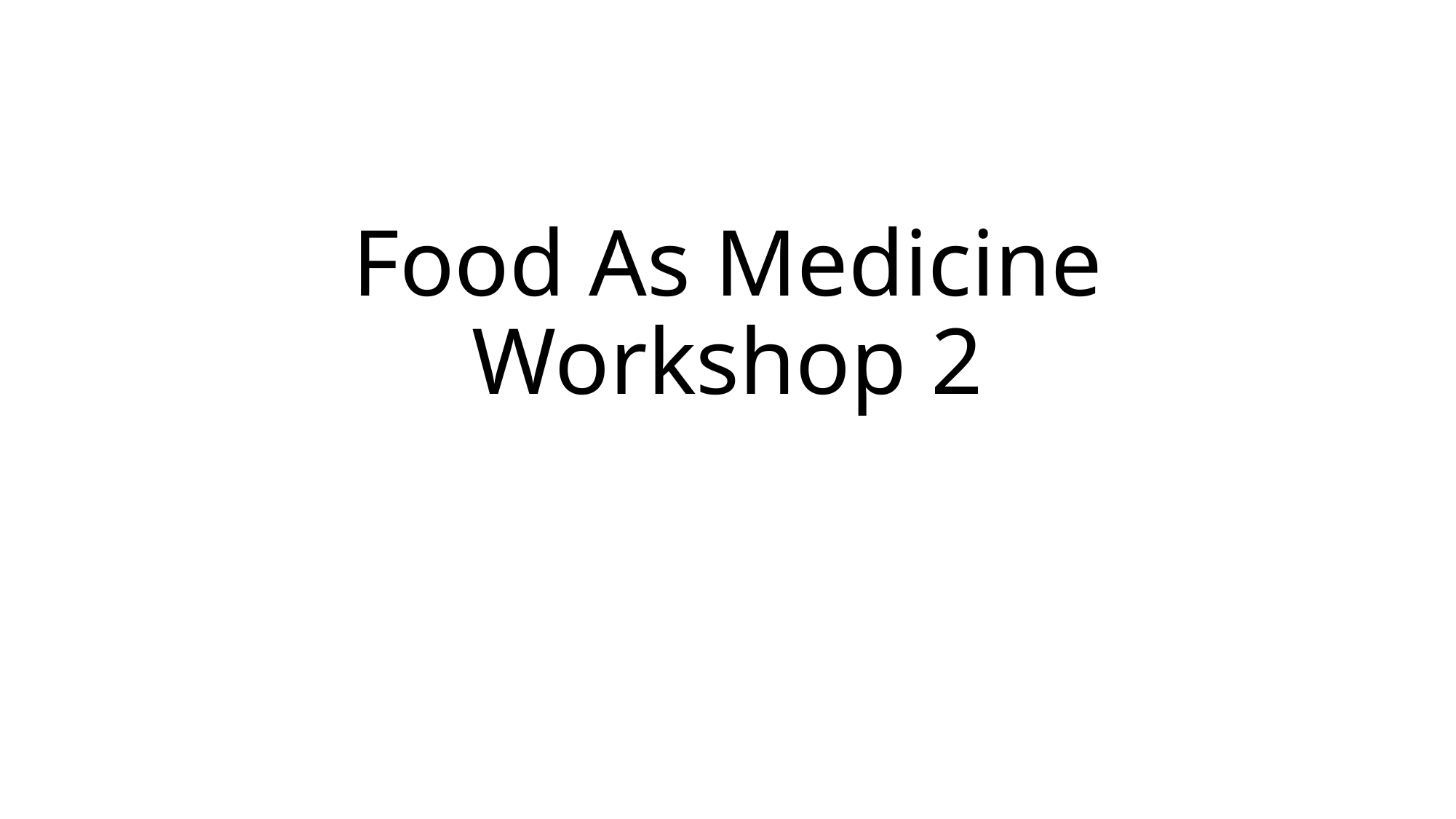

# Food As Medicine Workshop 2

## Slide 2
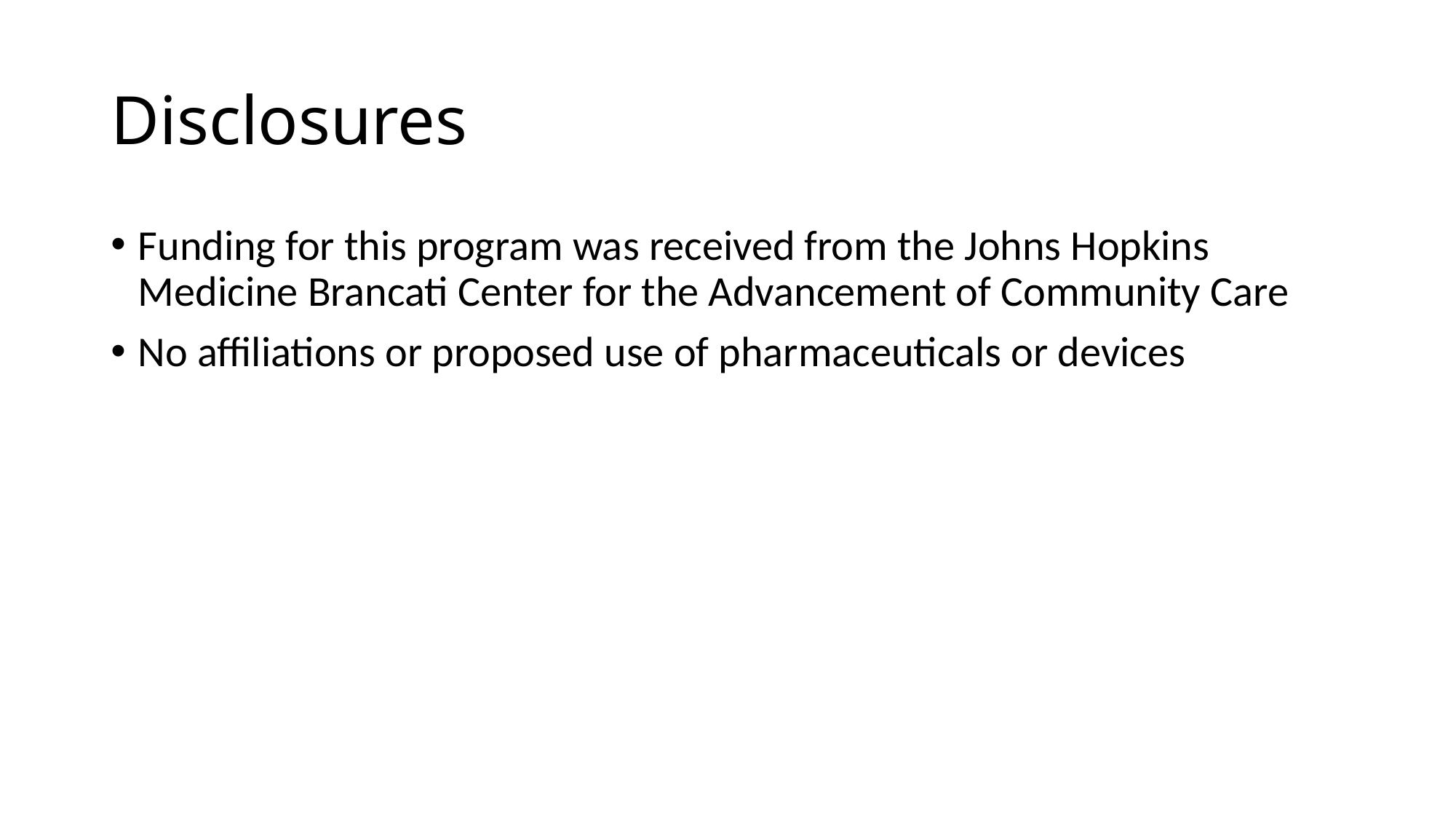

# Disclosures
Funding for this program was received from the Johns Hopkins Medicine Brancati Center for the Advancement of Community Care
No affiliations or proposed use of pharmaceuticals or devices

## Slide 3
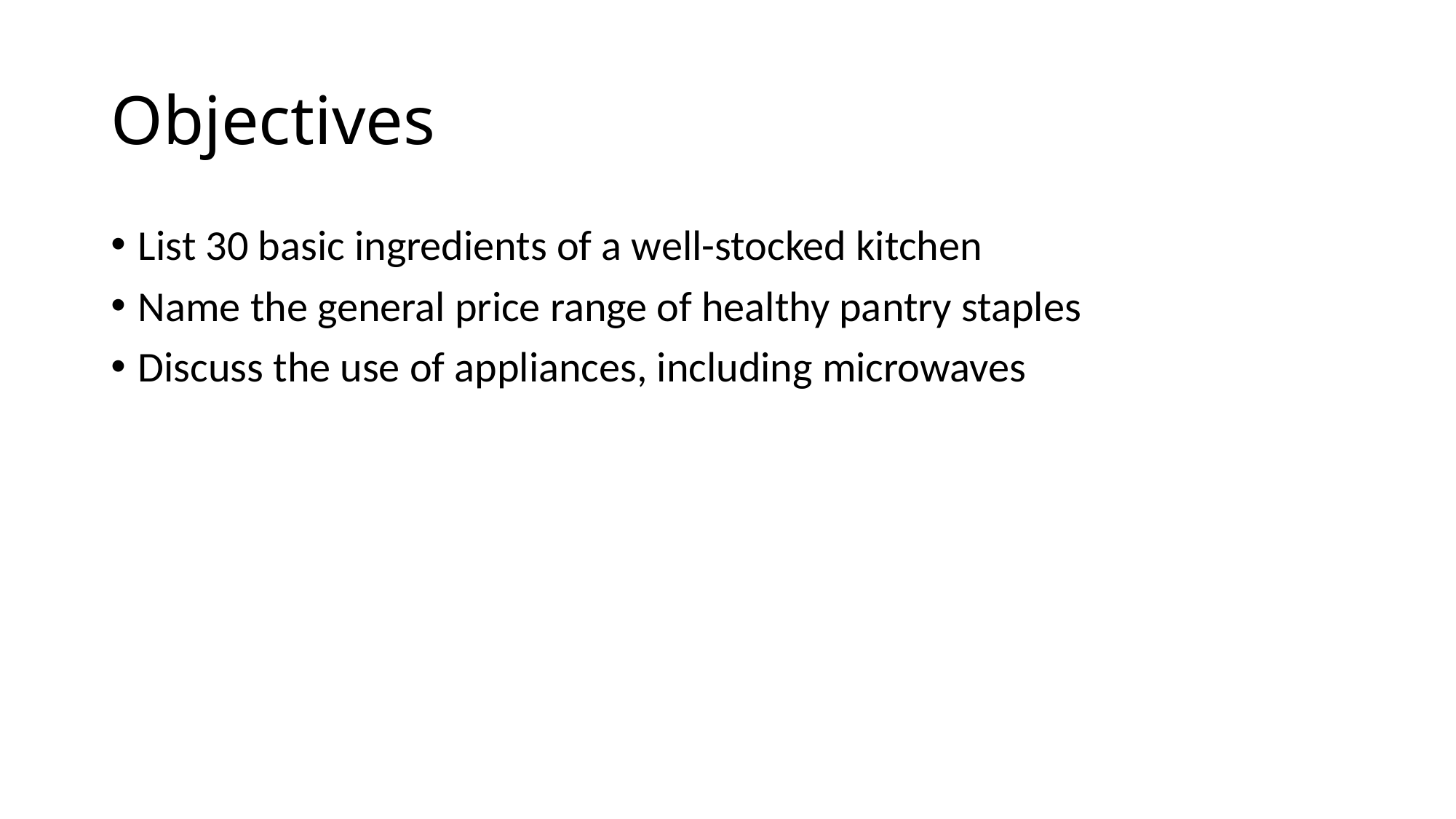

# Objectives
List 30 basic ingredients of a well-stocked kitchen
Name the general price range of healthy pantry staples
Discuss the use of appliances, including microwaves

## Slide 4
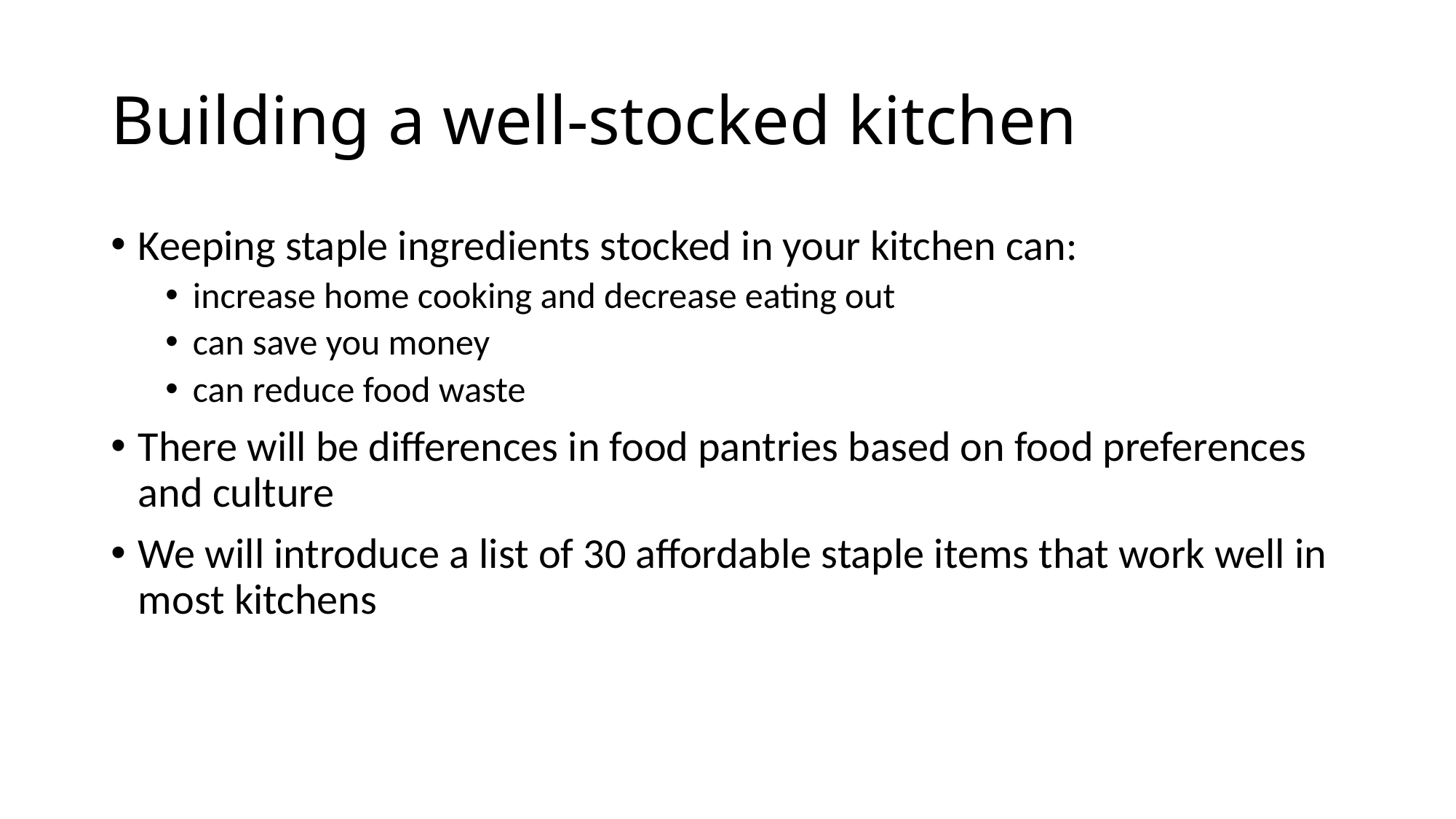

# Building a well-stocked kitchen
Keeping staple ingredients stocked in your kitchen can:
increase home cooking and decrease eating out
can save you money
can reduce food waste
There will be differences in food pantries based on food preferences and culture
We will introduce a list of 30 affordable staple items that work well in most kitchens

## Slide 5
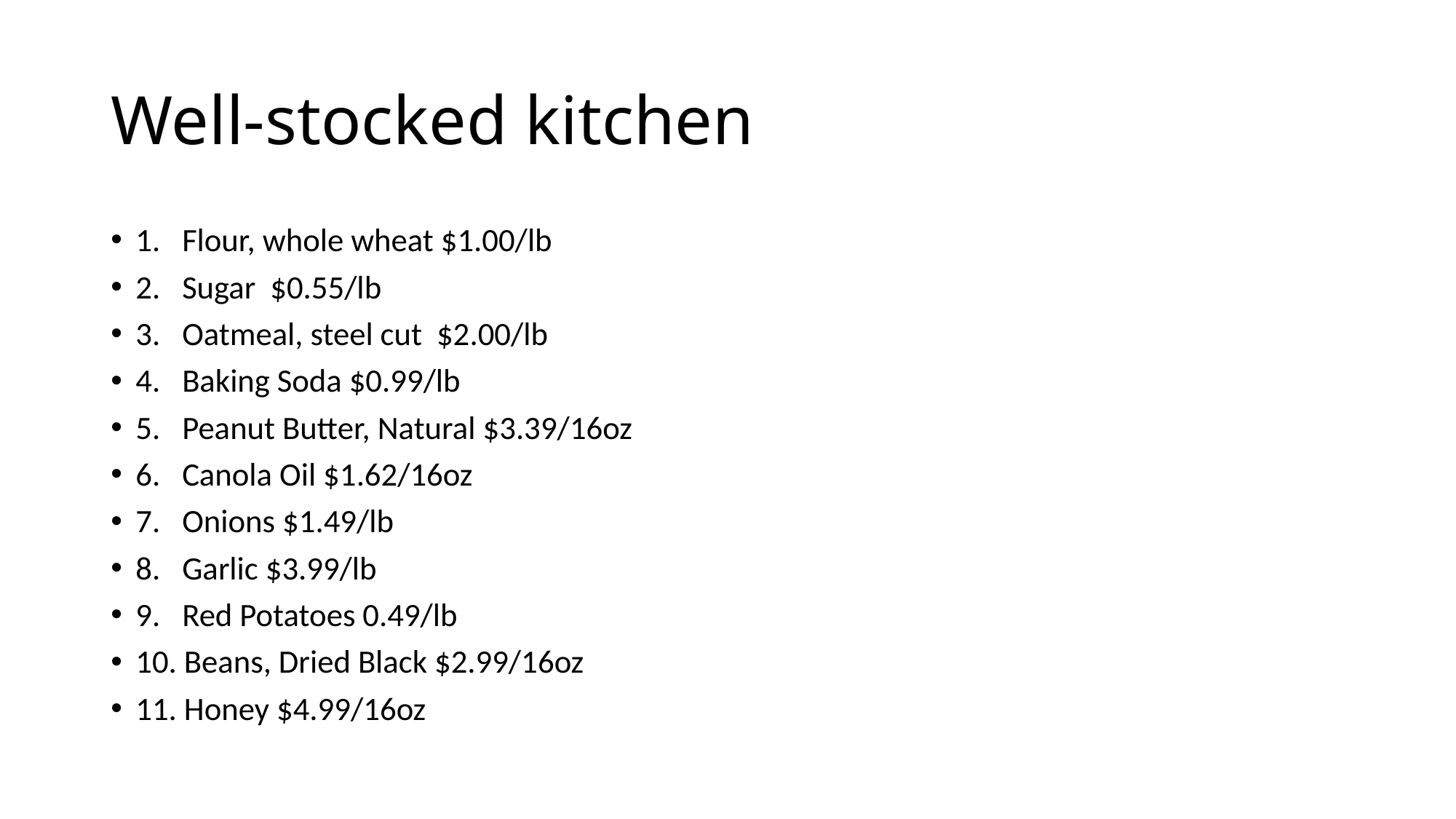

# Well-stocked kitchen
1. Flour, whole wheat $1.00/lb
2. Sugar $0.55/lb
3. Oatmeal, steel cut $2.00/lb
4. Baking Soda $0.99/lb
5. Peanut Butter, Natural $3.39/16oz
6. Canola Oil $1.62/16oz
7. Onions $1.49/lb
8. Garlic $3.99/lb
9. Red Potatoes 0.49/lb
10. Beans, Dried Black $2.99/16oz
11. Honey $4.99/16oz

## Slide 6
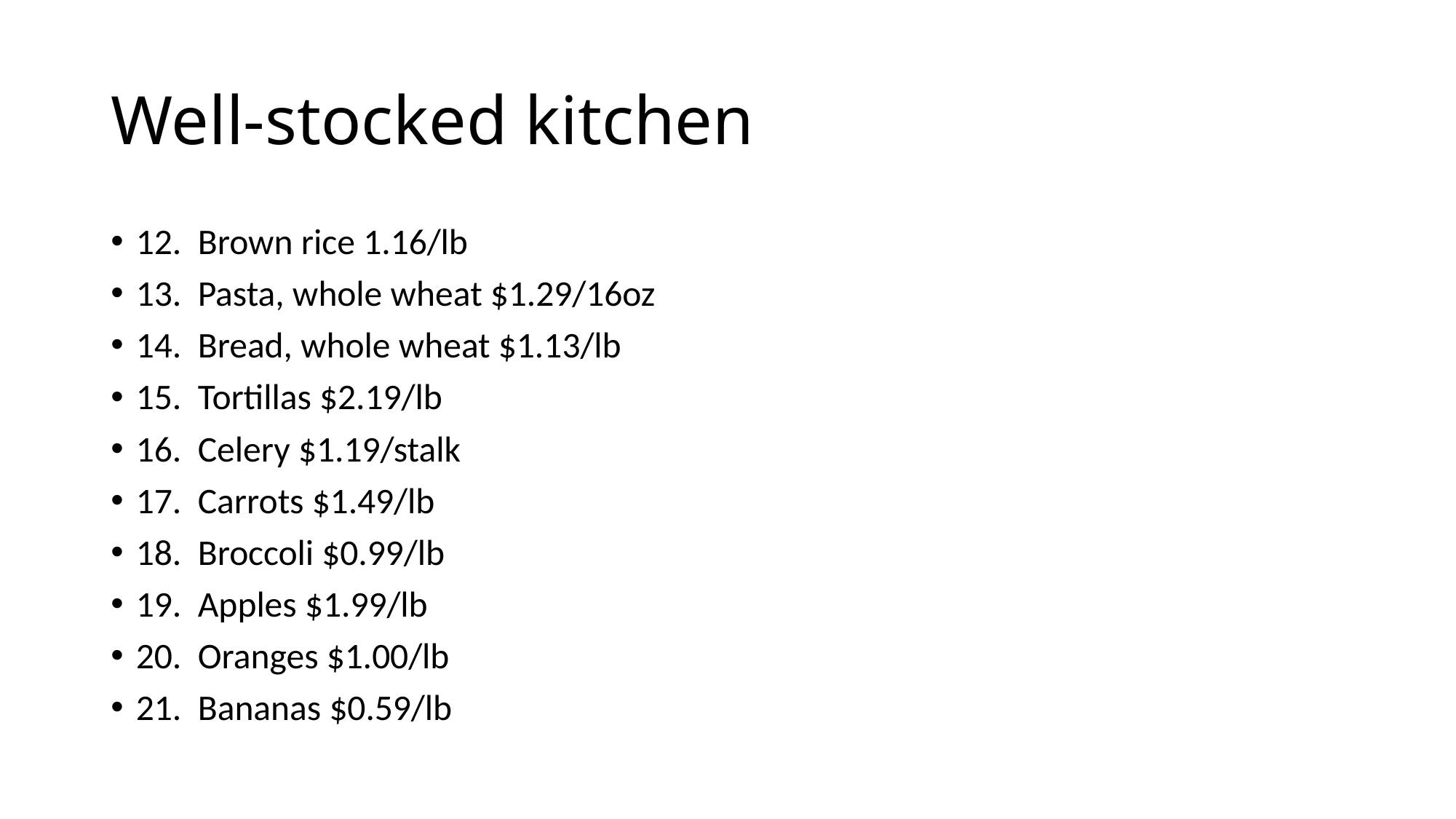

# Well-stocked kitchen
12. Brown rice 1.16/lb
13. Pasta, whole wheat $1.29/16oz
14. Bread, whole wheat $1.13/lb
15. Tortillas $2.19/lb
16. Celery $1.19/stalk
17. Carrots $1.49/lb
18. Broccoli $0.99/lb
19. Apples $1.99/lb
20. Oranges $1.00/lb
21. Bananas $0.59/lb

## Slide 7
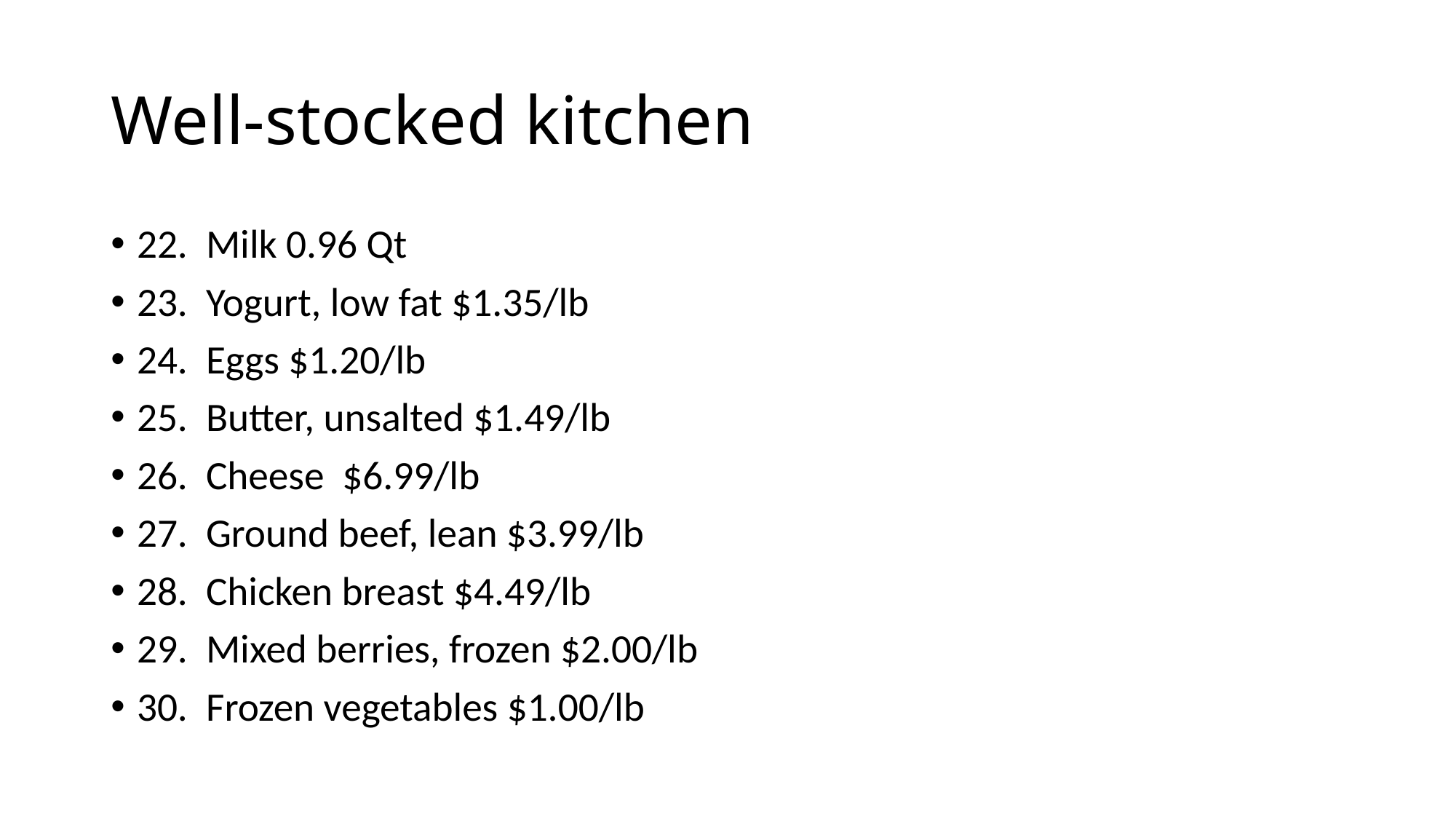

# Well-stocked kitchen
22. Milk 0.96 Qt
23. Yogurt, low fat $1.35/lb
24. Eggs $1.20/lb
25. Butter, unsalted $1.49/lb
26. Cheese $6.99/lb
27. Ground beef, lean $3.99/lb
28. Chicken breast $4.49/lb
29. Mixed berries, frozen $2.00/lb
30. Frozen vegetables $1.00/lb

## Slide 8
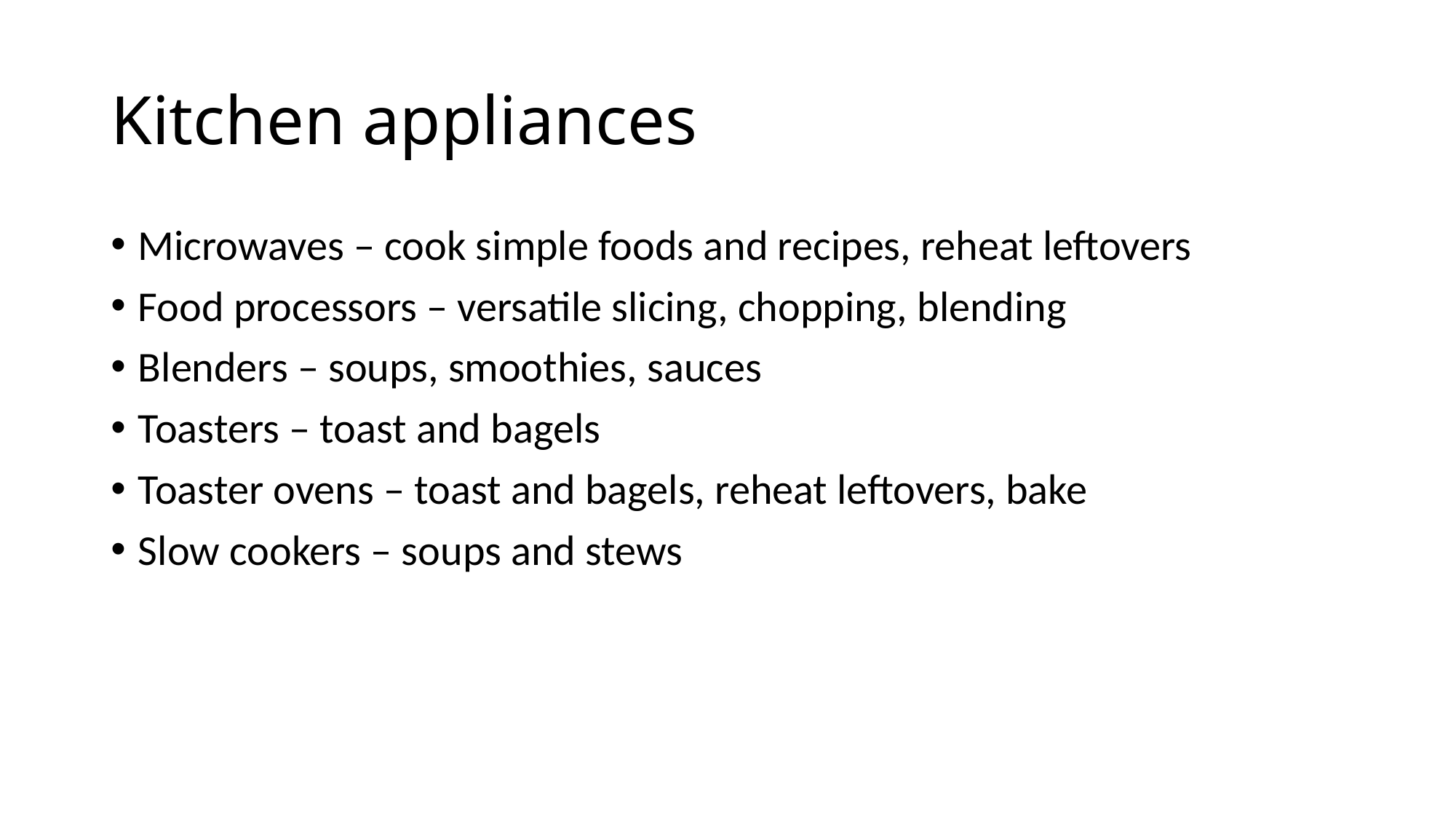

# Kitchen appliances
Microwaves – cook simple foods and recipes, reheat leftovers
Food processors – versatile slicing, chopping, blending
Blenders – soups, smoothies, sauces
Toasters – toast and bagels
Toaster ovens – toast and bagels, reheat leftovers, bake
Slow cookers – soups and stews

## Slide 9
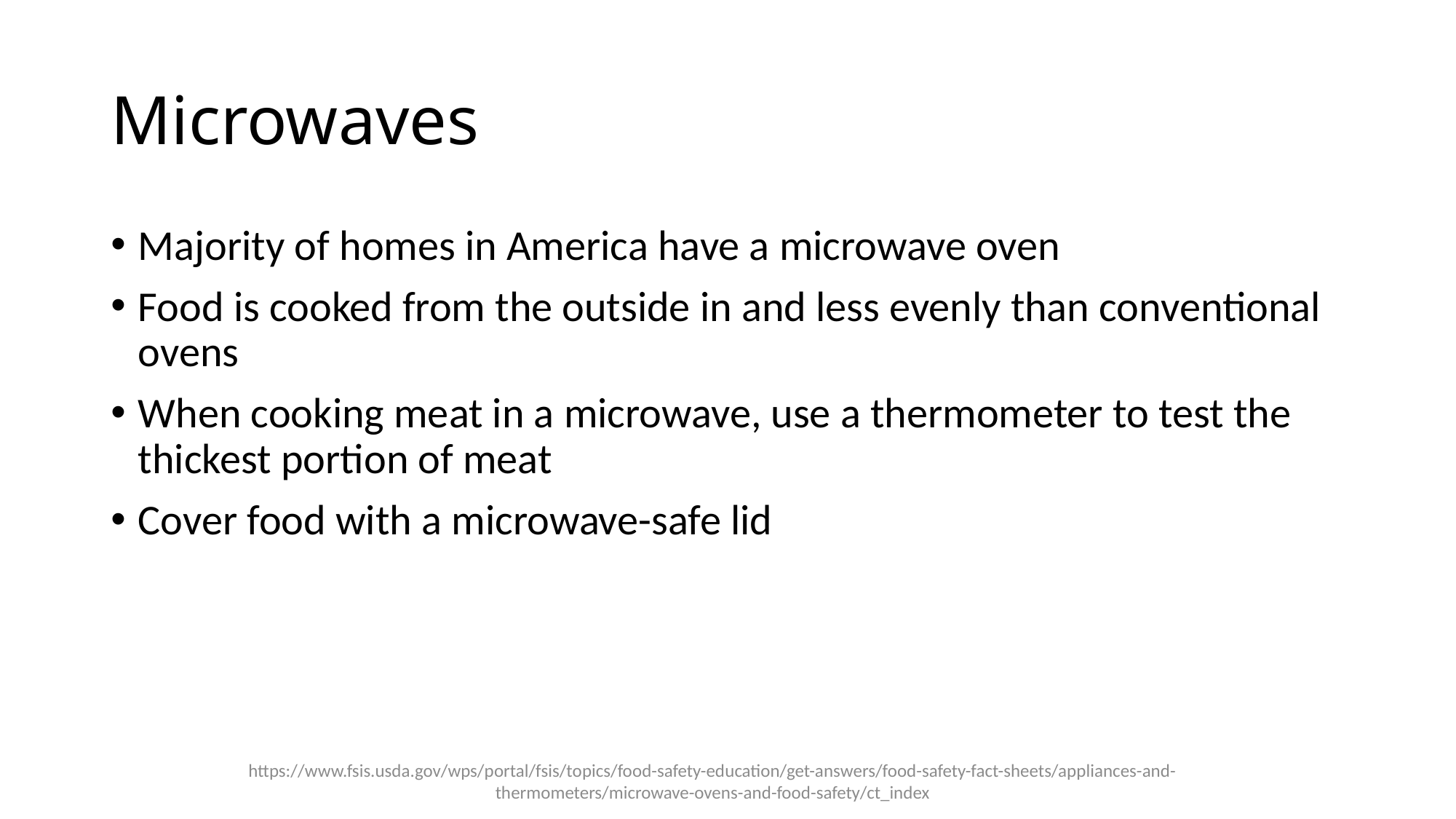

# Microwaves
Majority of homes in America have a microwave oven
Food is cooked from the outside in and less evenly than conventional ovens
When cooking meat in a microwave, use a thermometer to test the thickest portion of meat
Cover food with a microwave-safe lid
https://www.fsis.usda.gov/wps/portal/fsis/topics/food-safety-education/get-answers/food-safety-fact-sheets/appliances-and-thermometers/microwave-ovens-and-food-safety/ct_index

## Slide 10
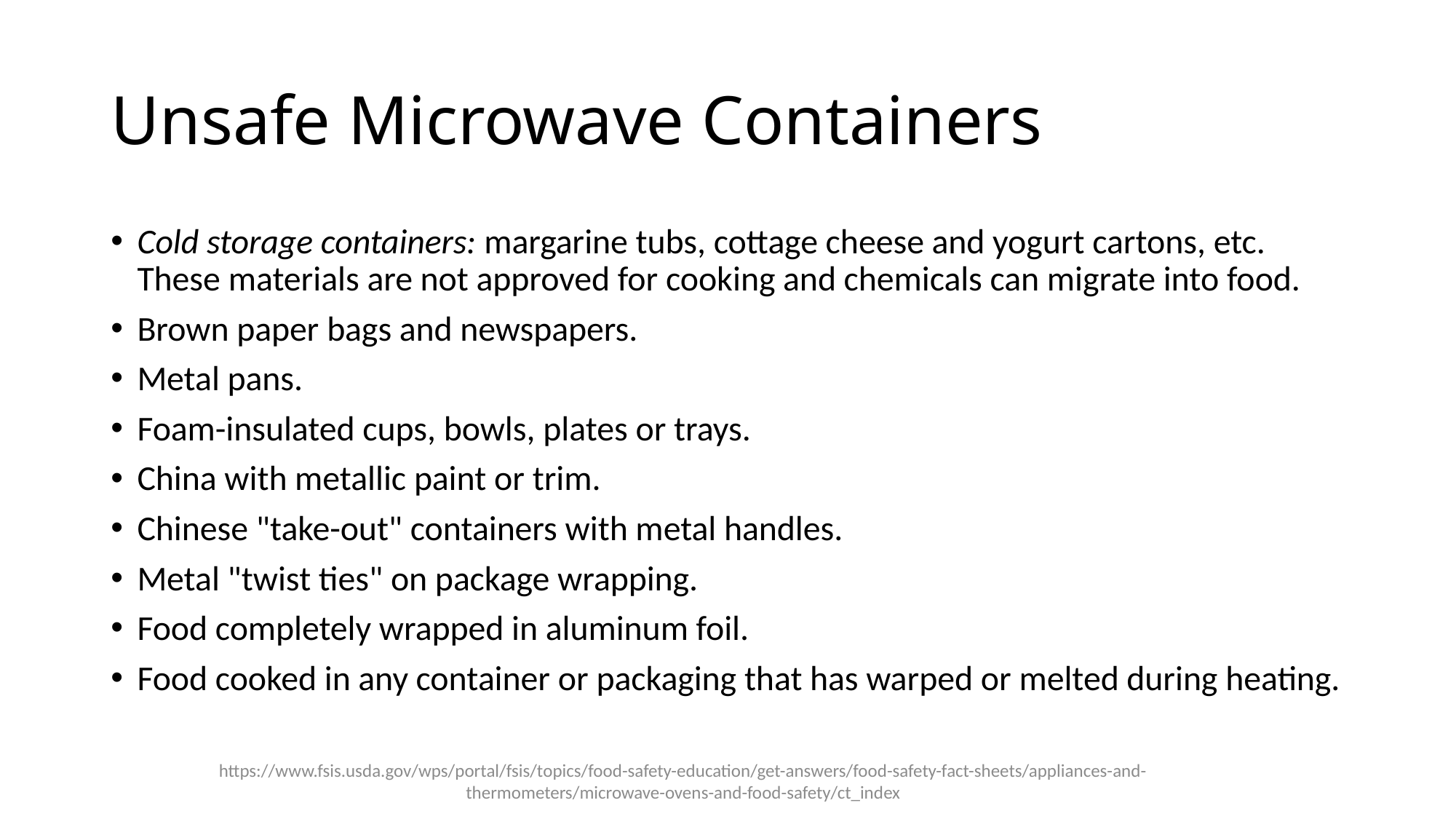

# Unsafe Microwave Containers
Cold storage containers: margarine tubs, cottage cheese and yogurt cartons, etc. These materials are not approved for cooking and chemicals can migrate into food.
Brown paper bags and newspapers.
Metal pans.
Foam-insulated cups, bowls, plates or trays.
China with metallic paint or trim.
Chinese "take-out" containers with metal handles.
Metal "twist ties" on package wrapping.
Food completely wrapped in aluminum foil.
Food cooked in any container or packaging that has warped or melted during heating.
https://www.fsis.usda.gov/wps/portal/fsis/topics/food-safety-education/get-answers/food-safety-fact-sheets/appliances-and-thermometers/microwave-ovens-and-food-safety/ct_index
