# Supplementary material for: Community Culinary Workshops as a Nutrition Curriculum in a Preventive Medicine Residency Program
Source: MedEdPORTAL. 2019 Dec 13;15:10859. doi: 10.15766/mep_2374-8265.10859 (PMC7010195; doi:10.15766/mep_2374-8265.10859)
Supplement: Supplementary file 1 — A. Facilitator Guide.docx B. Workshop 1 Presentation.pptx C. Workshop 2 Presentation.pptx D. Workshop 3 Presentation.pptx E. Tofu Lettuce Cups Recipe.pdf F. Kale Pesto Recipe.pdf G. Cold Asian Noodles Recipe.pdf H. Postworkshop Survey.docx [file mep-15-10859-s001.zip › D. Workshop 3 Presentation.pptx]

## Slide 1
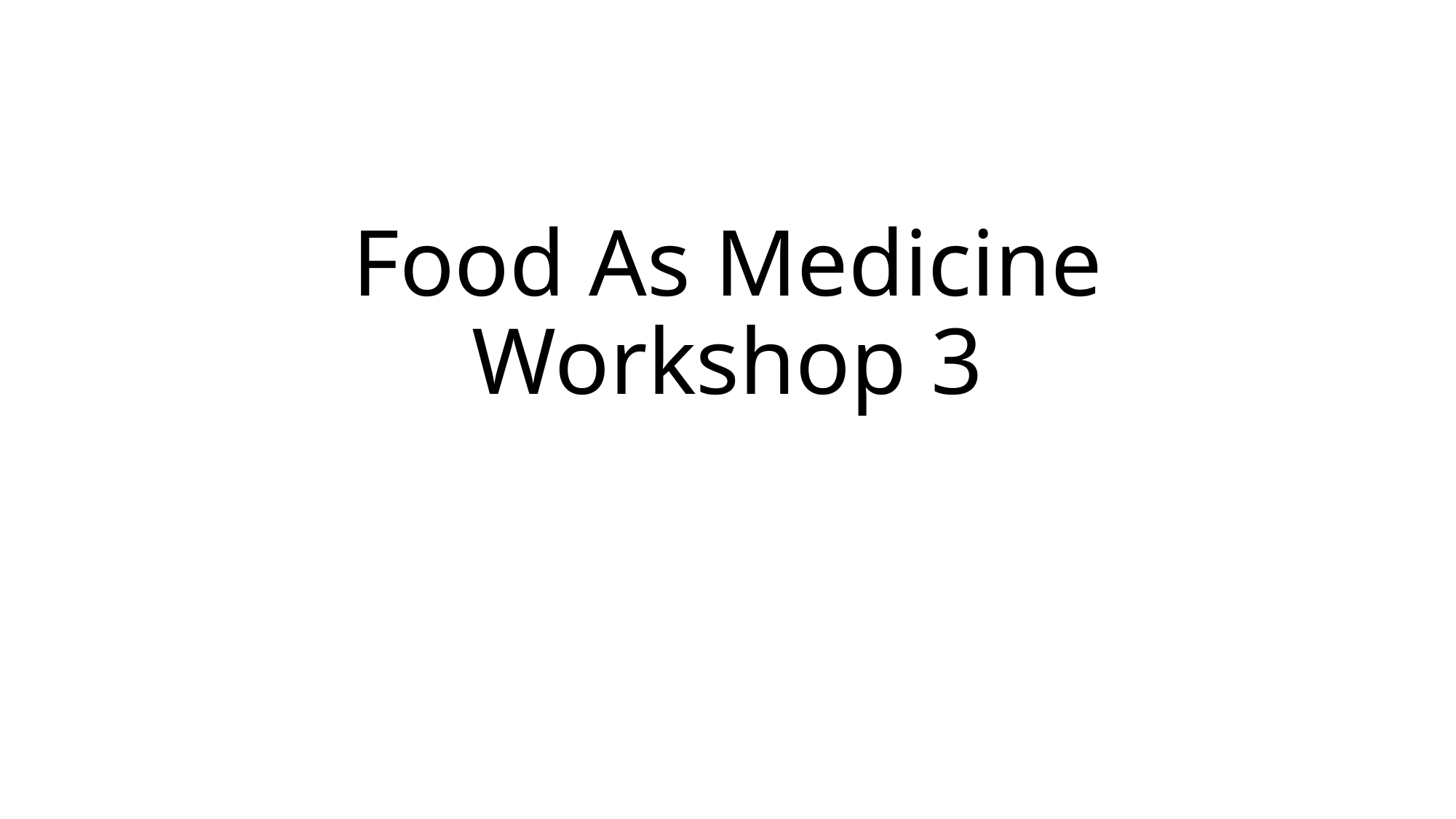

# Food As Medicine Workshop 3

## Slide 2
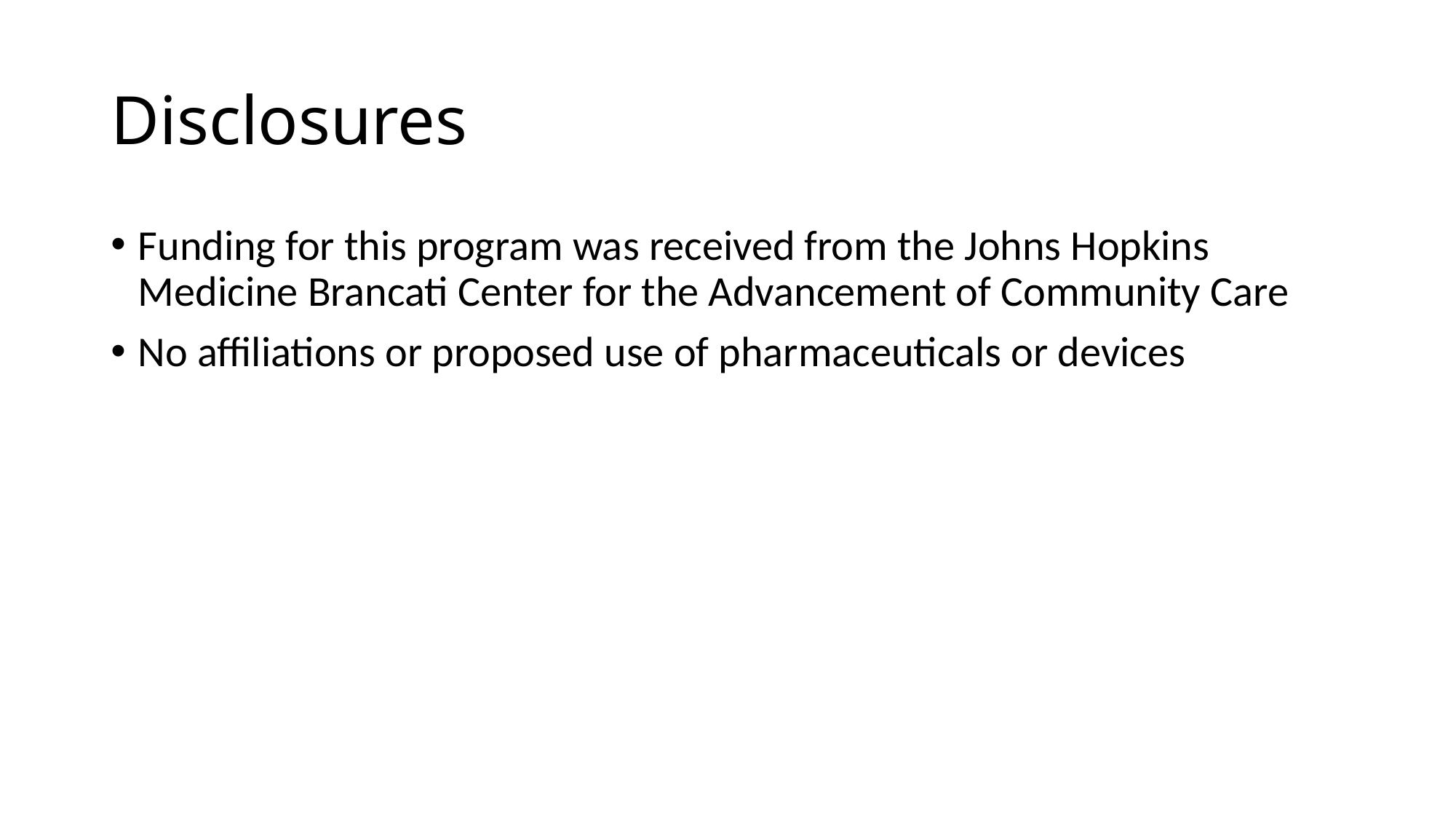

# Disclosures
Funding for this program was received from the Johns Hopkins Medicine Brancati Center for the Advancement of Community Care
No affiliations or proposed use of pharmaceuticals or devices

## Slide 3
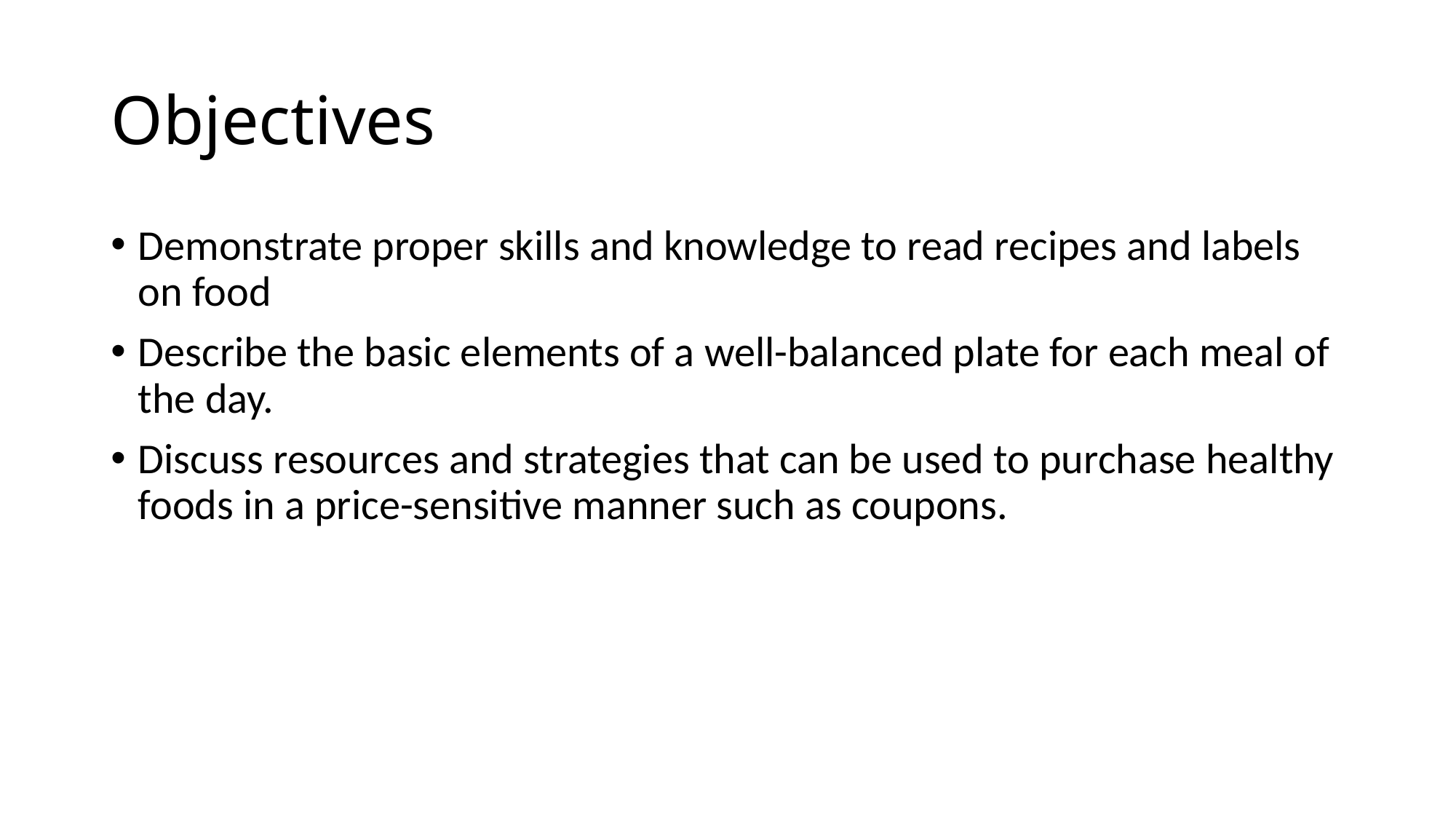

# Objectives
Demonstrate proper skills and knowledge to read recipes and labels on food
Describe the basic elements of a well-balanced plate for each meal of the day.
Discuss resources and strategies that can be used to purchase healthy foods in a price-sensitive manner such as coupons.

## Slide 4
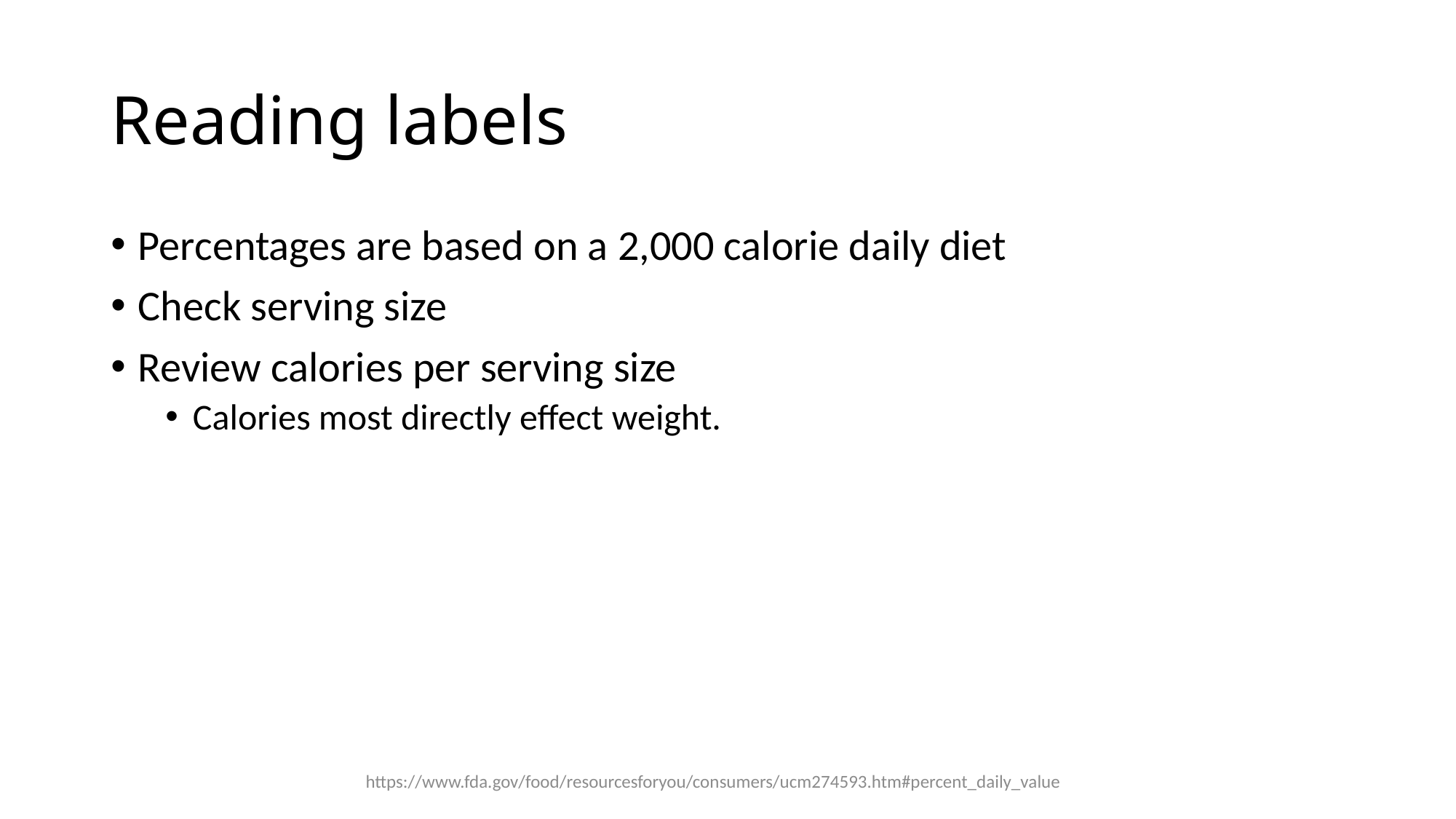

# Reading labels
Percentages are based on a 2,000 calorie daily diet
Check serving size
Review calories per serving size
Calories most directly effect weight.
https://www.fda.gov/food/resourcesforyou/consumers/ucm274593.htm#percent_daily_value

## Slide 5
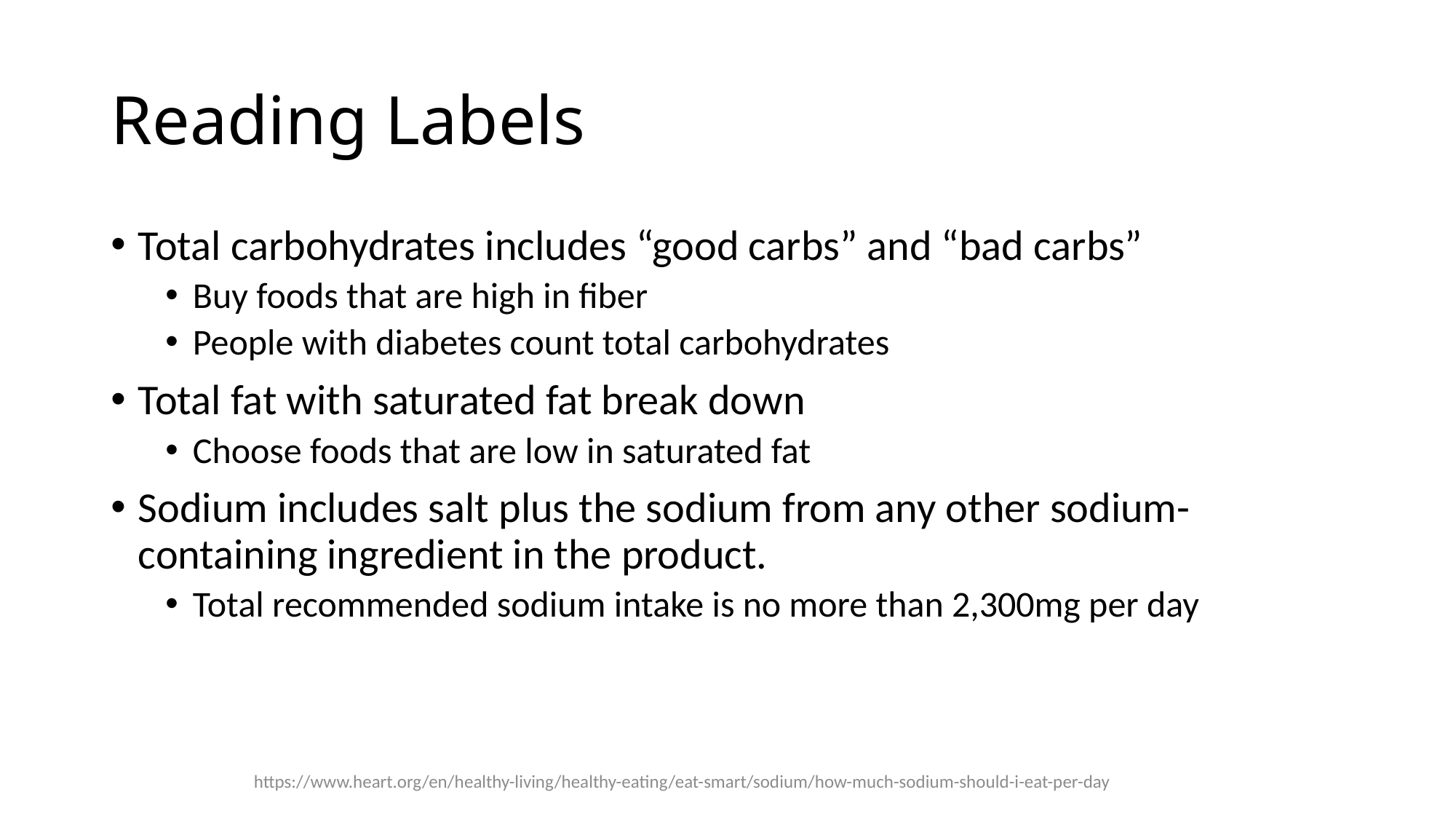

# Reading Labels
Total carbohydrates includes “good carbs” and “bad carbs”
Buy foods that are high in fiber
People with diabetes count total carbohydrates
Total fat with saturated fat break down
Choose foods that are low in saturated fat
Sodium includes salt plus the sodium from any other sodium-containing ingredient in the product.
Total recommended sodium intake is no more than 2,300mg per day
https://www.heart.org/en/healthy-living/healthy-eating/eat-smart/sodium/how-much-sodium-should-i-eat-per-day

## Slide 6
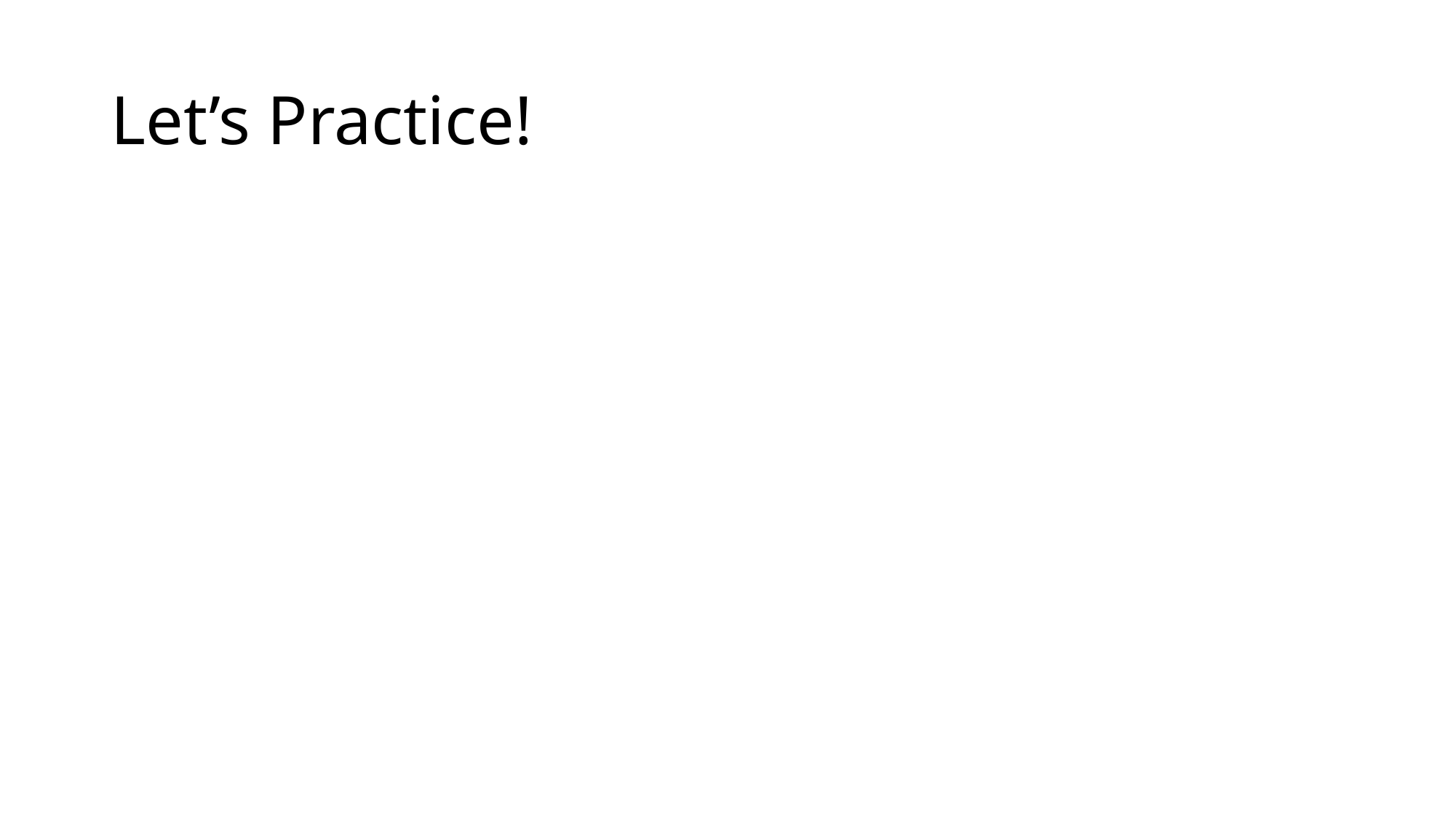

# Let’s Practice!

## Slide 7
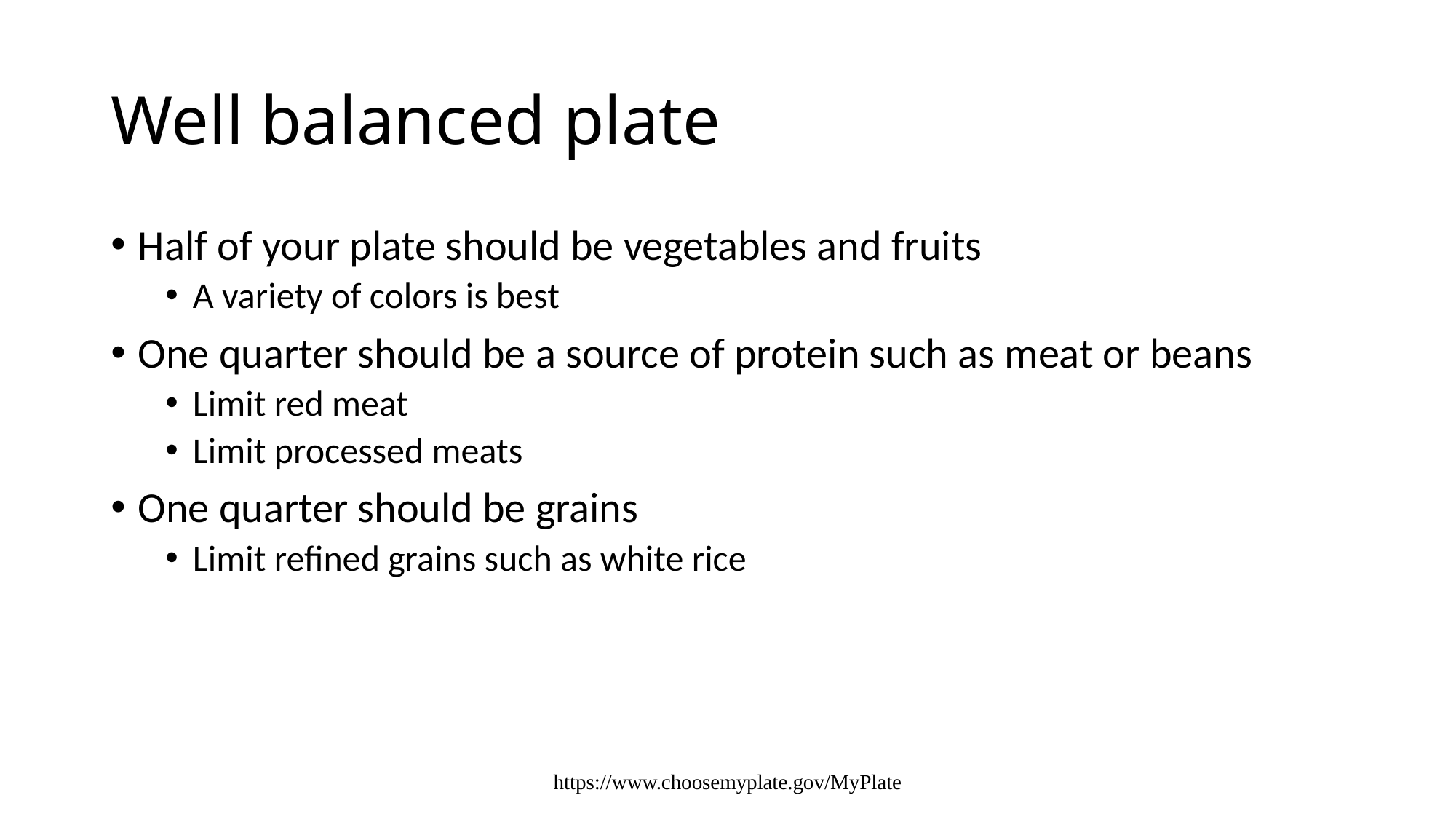

# Well balanced plate
Half of your plate should be vegetables and fruits
A variety of colors is best
One quarter should be a source of protein such as meat or beans
Limit red meat
Limit processed meats
One quarter should be grains
Limit refined grains such as white rice
https://www.choosemyplate.gov/MyPlate

## Slide 8
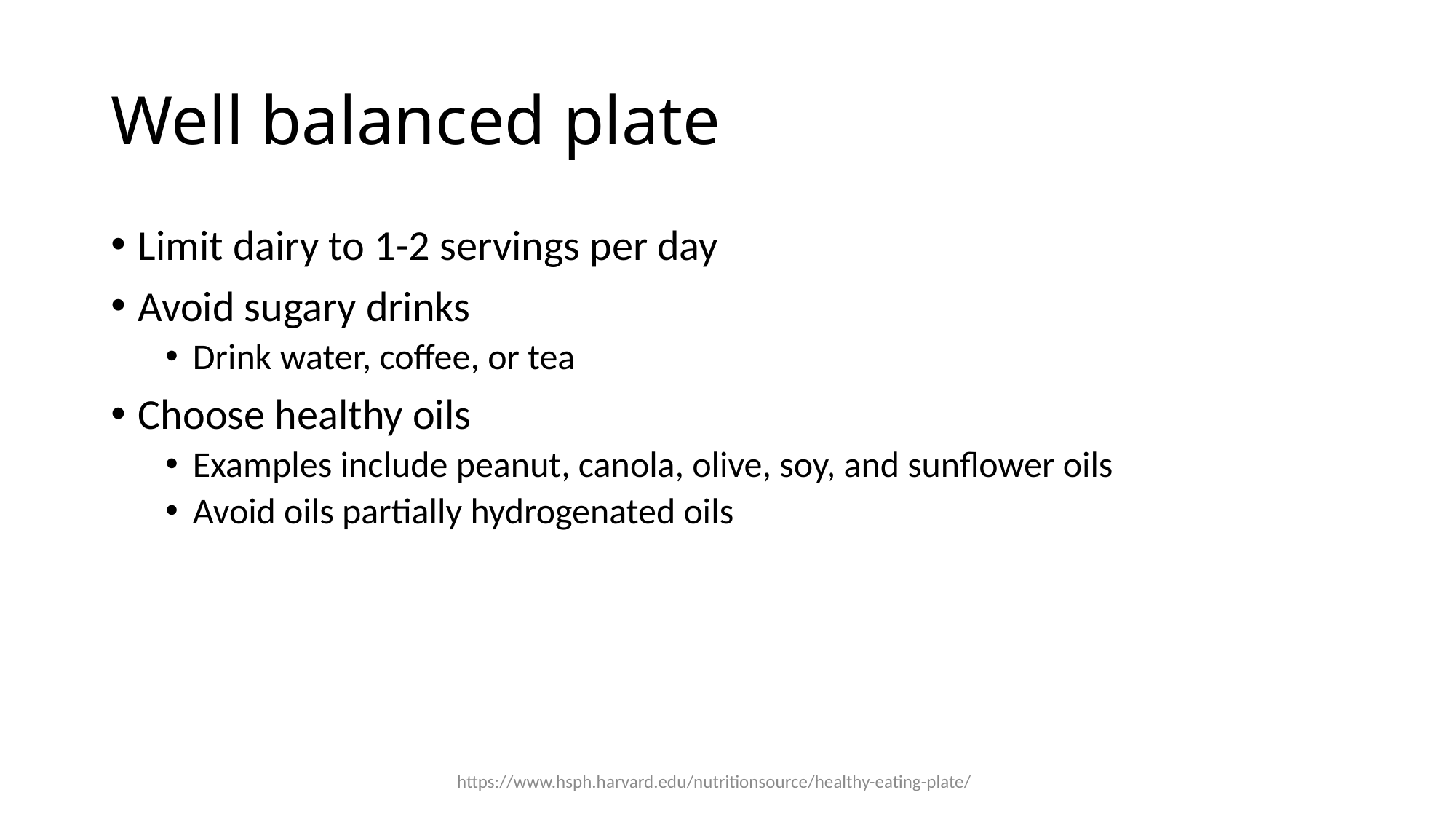

# Well balanced plate
Limit dairy to 1-2 servings per day
Avoid sugary drinks
Drink water, coffee, or tea
Choose healthy oils
Examples include peanut, canola, olive, soy, and sunflower oils
Avoid oils partially hydrogenated oils
https://www.hsph.harvard.edu/nutritionsource/healthy-eating-plate/

## Slide 9
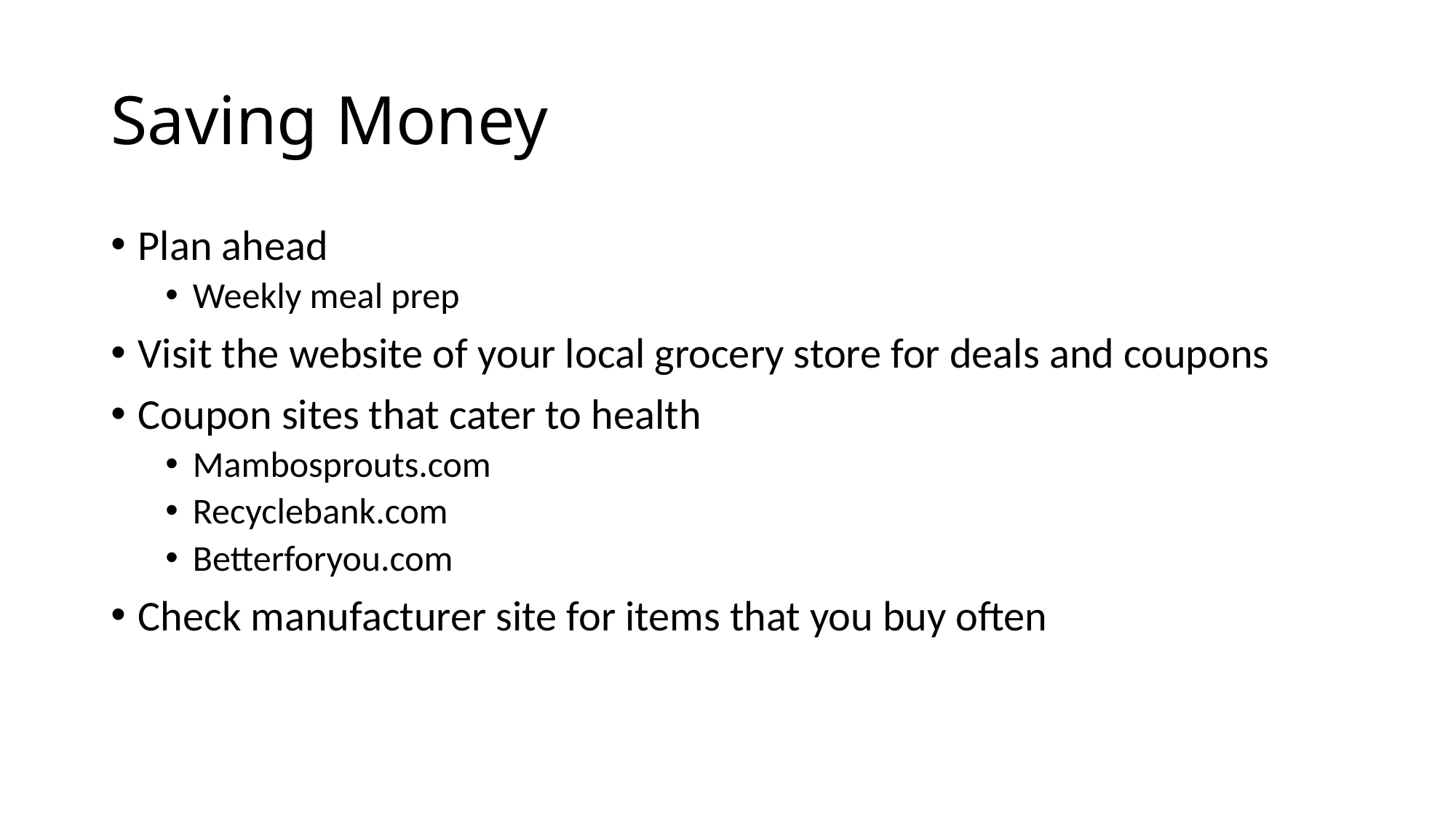

# Saving Money
Plan ahead
Weekly meal prep
Visit the website of your local grocery store for deals and coupons
Coupon sites that cater to health
Mambosprouts.com
Recyclebank.com
Betterforyou.com
Check manufacturer site for items that you buy often

## Slide 10
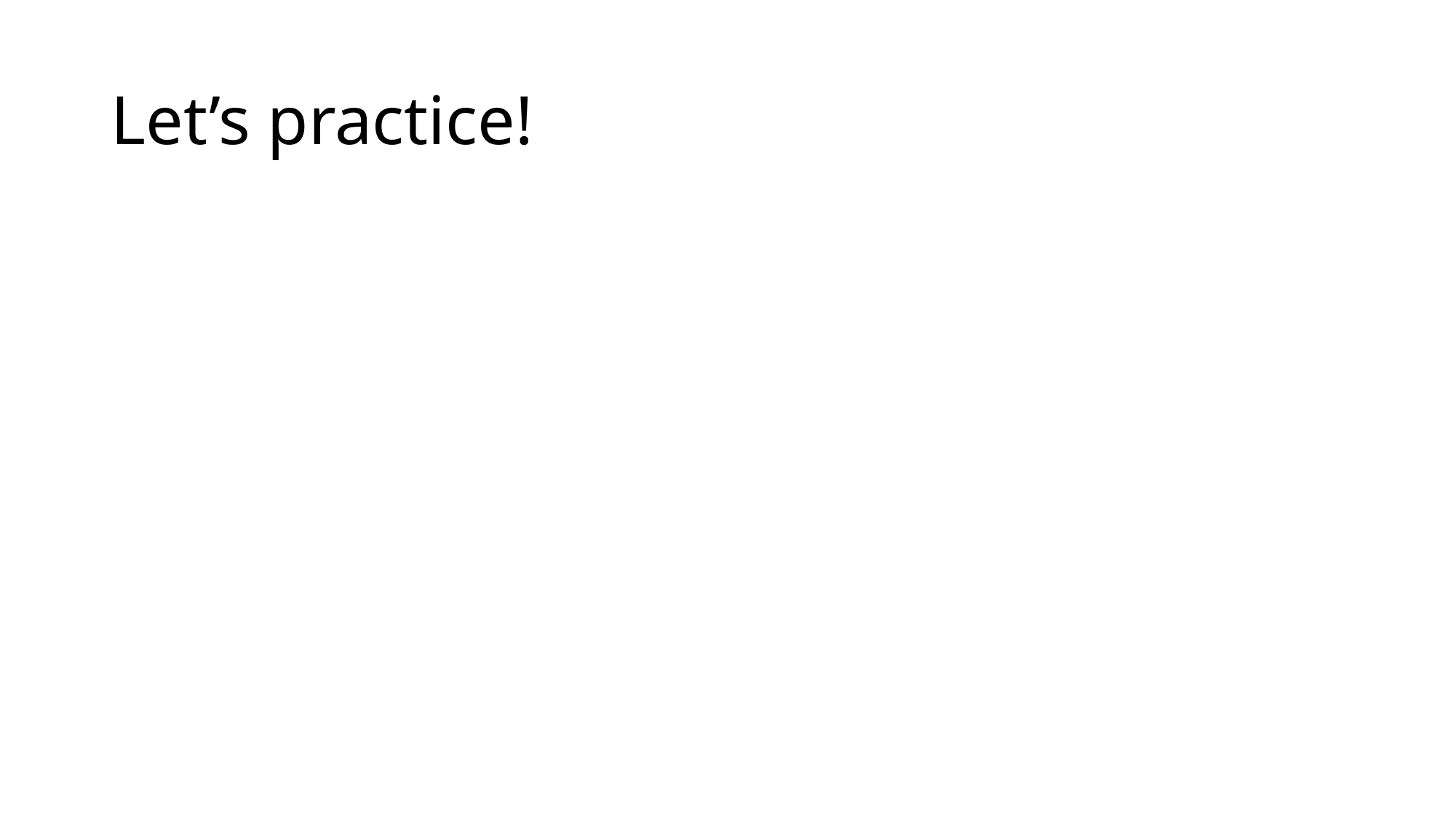

# Let’s practice!
