# Supplementary material for: Community Culinary Workshops as a Nutrition Curriculum in a Preventive Medicine Residency Program
Source: MedEdPORTAL. 2019 Dec 13;15:10859. doi: 10.15766/mep_2374-8265.10859 (PMC7010195; doi:10.15766/mep_2374-8265.10859)
Supplement: Supplementary file 1 — A. Facilitator Guide.docx B. Workshop 1 Presentation.pptx C. Workshop 2 Presentation.pptx D. Workshop 3 Presentation.pptx E. Tofu Lettuce Cups Recipe.pdf F. Kale Pesto Recipe.pdf G. Cold Asian Noodles Recipe.pdf H. Postworkshop Survey.docx [file mep-15-10859-s001.zip › E. Tofu Lettuce Cups Recipe.pdf]

Tofu Lettuce Cups
Serving Size: 8 Servings
Ingredients:

1 teaspoon kosher salt
1 teaspoon fresh cracked pepper
16 butter lettuce leaves
1 tablespoon plus 1 teaspoons expeller-pressed canola oil (non GMO)
12 ounces extra-firm organic tofu, cubed
6 ounces shiitake mushrooms, stemmed and sliced (white button/portobella_optional)
1 tablespoon fresh ginger, grated
6 scallions, whites only, chopped
1/4 cup low-sodium soy sauce (tamari optional)
1/4 cup freshly squeezed orange juice
6 scallions, greens-only, chopped
1 cup diced jicama, peeled (optional)
2 ¼ teaspoons toasted sesame seeds (optional)
1 carrot, shaved into ribbons with vegetable peeler, or can use shredded carrot

Equipment:
1 skillet
1 heat-resistant spatula
2 rectangular serving plates or platters

Procedure:
1. Take two leaves and nestle one on top of another to form one cup. Place on a platter and do this for the remaining leaves for a total of 8 cups.
2. In a skillet, heat the oil on medium heat until it shimmers. Add the tofu, mushrooms into the pan. Grate the ginger into the mixture and add the whites-only scallions into the skillet. Cook until tofu is light brown, stir occasionally. Cook for 10 minutes.
3. Add the tamari and fresh orange juice. Cook for another 5 minutes until sauce thickens. Then add the diced jicama and 1 ½ teaspoons of sesame seeds and cook for another 2 minutes.
4. Use a slotted spoon to ladle the mixture into each lettuce cup.
5. Sprinkle shredded carrot and scallions (greens-only) and remaining sesame seeds into each cup. Divide appropriately.
6. Serve immediately.

~ Colin Zhu, DO 2016
