# Supplementary material for: Community Culinary Workshops as a Nutrition Curriculum in a Preventive Medicine Residency Program
Source: MedEdPORTAL. 2019 Dec 13;15:10859. doi: 10.15766/mep_2374-8265.10859 (PMC7010195; doi:10.15766/mep_2374-8265.10859)
Supplement: Supplementary file 1 — A. Facilitator Guide.docx B. Workshop 1 Presentation.pptx C. Workshop 2 Presentation.pptx D. Workshop 3 Presentation.pptx E. Tofu Lettuce Cups Recipe.pdf F. Kale Pesto Recipe.pdf G. Cold Asian Noodles Recipe.pdf H. Postworkshop Survey.docx [file mep-15-10859-s001.zip › F. Kale Pesto Recipe.pdf]

Kale Pesto
Serving Size: 8
Yield: 2 cups

Ingredients:
8 cups stemmed chopped organic kale (~ 2 bunches)
3/4 cup extra virgin olive oil
1/2 cup walnuts or (pine nuts_optional)
4 garlic cloves, chopped
2 teaspoons kosher salt
1/2 teaspoon red pepper flakes (optional)
1/4 cup chia seeds (optional)
French whole wheat baguette or
Whole wheat pasta (optional)

Equipment:
Ice bath
colander
food processor

Procedure:
1. Bring a large pot of water to a boil. Fill a separate bowl with ice cubes and cold water.
2. Plunge the kale into the boiling water for 3 mins. Using tongs, transfer the kale from the hot water to the ice bath (blanch and shock). After 3 mins, drain the kale in a colander, then squeeze it firmly to press out excess water.
3. Put kale and all of the remaining ingredients in a food processor and puree until smooth. Transfer to a serving platter with baguette, sliced. Spread pesto over each bread slice and serve. Or, transfer to a container, cover and refrigerate until ready to use. Pesto will keep up to 3 days.

~ Colin Zhu, D.O. 2016
