# Supplementary material for: Community Culinary Workshops as a Nutrition Curriculum in a Preventive Medicine Residency Program
Source: MedEdPORTAL. 2019 Dec 13;15:10859. doi: 10.15766/mep_2374-8265.10859 (PMC7010195; doi:10.15766/mep_2374-8265.10859)
Supplement: Supplementary file 1 — A. Facilitator Guide.docx B. Workshop 1 Presentation.pptx C. Workshop 2 Presentation.pptx D. Workshop 3 Presentation.pptx E. Tofu Lettuce Cups Recipe.pdf F. Kale Pesto Recipe.pdf G. Cold Asian Noodles Recipe.pdf H. Postworkshop Survey.docx [file mep-15-10859-s001.zip › G. Cold Asian Noodles Recipe.pdf]

Cold Glass Asian Noodles with Ginger Cilantro Mint Sauce
Serving Size: 4

Ginger Cilantro Mint Sauce:
1/2 inch fresh ginger, minced
1 tbsp fresh cilantro, minced
1 tbsp fresh mint, minced
4 cloves of garlic, minced
8 oz of low sodium soy sauce or Tamari
1 tsp sesame oil
2 tsp rice vinegar

Ingredients:
2 packages of mung bean noodles
1 red bell pepper, sliced
1 yellow bell pepper, sliced
2.5 oz of shiitake mushrooms, dried
1/2 medium size jicama, peeled and sliced
romaine lettuce, sliced

Equipment:
1 medium bowl
1 ice bath

Procedure:
1. Use medium size skillet or large sauce pot, fill and start to boil water.
2. While waiting to boil, combine ingredients for sauce and sit aside.
3. When water is boiling, place six mushrooms into water and let rehydrate and cook, then once soft (3 minutes), take out and slice and place aside to cool.
4. Place noodles into boiling water and let cook down. Add more water if necessary. Stir occasionally. While cooking down, prepare other vegetables. Set aside.
5. Once noodles are cooked down, place into ice bath for 30 seconds or so [or alternative is rinse through colander under cold water.] Serve into four bowls, rinsed off of water. Then add vegetables on top with mushrooms. Serve with sauce.
6. Serve immediately cold.

~ Colin Zhu, DO 2017
