# Supplementary material for: Community Culinary Workshops as a Nutrition Curriculum in a Preventive Medicine Residency Program
Source: MedEdPORTAL. 2019 Dec 13;15:10859. doi: 10.15766/mep_2374-8265.10859 (PMC7010195; doi:10.15766/mep_2374-8265.10859)
Supplement: Supplementary file 1 — A. Facilitator Guide.docx B. Workshop 1 Presentation.pptx C. Workshop 2 Presentation.pptx D. Workshop 3 Presentation.pptx E. Tofu Lettuce Cups Recipe.pdf F. Kale Pesto Recipe.pdf G. Cold Asian Noodles Recipe.pdf H. Postworkshop Survey.docx [file mep-15-10859-s001.zip › H. Postworkshop Survey.docx]

Thank you for your willingness to complete this survey.

You are being asked to take this survey as a participant in a series of community cooking classes. Your participation in this survey is entirely voluntary. It will take approximately 10 minutes to complete.

Thank you for your time and participation.

Date: _________

Please describe your role:

Medical Assistant

Nurse

Preventive Medicine Resident

Other

How long have you been in your current position

Less than 5 years

Between 5 and 10 years

Between 11 and 15 years

Between 16 and 20 years

Greater than 20 years

***The following questions are used to gather primary outcomes for the study.***

How many times per week do you eat home cooked meals on average?

How many times week do you cook at home on average?

How many fruits per day do you eat on average?

How many vegetables per day do you eat on average?

*Please indicate your level of agreement or disagreement with the following statements.*

I feel comfortable counseling patients about cooking, eating and nutrition

Strongly Disagree Disagree Neutral Agree Strongly Agree

I myself am a competent cook

Strongly Disagree Disagree Neutral Agree Strongly Agree

Have you taken a cooking workshop before? Yes No

If yes, how many?

***The following questions are used to gather secondary outcomes for the study.***

I feel comfortable counseling patients about Integrative/Lifestyle Medicine

Strongly Disagree Disagree Neutral Agree Strongly Agree

Most patients will try to change their lifestyle if I advise them to do so

Strongly Disagree Disagree Neutral Agree Strongly Agree

Physicians can have an effect on a patient’s dietary behavior if they take the time to discuss the problem

Strongly Disagree Disagree Neutral Agree Strongly Agree

Clinic staff can have an effect on a patient’s dietary behavior if they take the time to discuss the problem

Strongly Disagree Disagree Neutral Agree Strongly Agree

For most patients, health education does little to promote adherence to a healthy lifestyle

Strongly Disagree Disagree Neutral Agree Strongly Agree

After receiving nutrition counseling, patient with poor habits will make major changes in their eating behavior.

Strongly Disagree Disagree Neutral Agree Strongly Agree

My patient-education efforts will be effective in increasing patients’ compliance with nutritional recommendations.

Strongly Disagree Disagree Neutral Agree Strongly Agree

After receiving nutritional counseling, patients with poor eating habits will make moderate changes in their eating behavior.

Strongly Disagree Disagree Neutral Agree Strongly Agree

I feel comfortable prescribing nutritional interventions for disease management

Strongly Disagree Disagree Neutral Agree Strongly Agree

How much does your knowledge of Integrative Medicine/Lifestyle Medicine impact the type of care that you provide for your patients?

Not at all  Very slightly  Moderately  Significantly  Unsure
